# Trematode Infection Prevalence Increases With Snail Richness: Observations From a 4‐Year Study of Snail–Trematode Interactions

**DOI:** 10.1002/ece3.72381

**Published:** 2025-10-22

**Authors:** Brooke A. McPhail, Carol M. Frost, Simon J. G. Otto, Patrick C. Hanington

**Affiliations:** ^1^ School of Public Health University of Alberta Edmonton Alberta Canada; ^2^ Department of Renewable Resources University of Alberta Edmonton Alberta Canada; ^3^ Human‐Environment‐Animal Transdisciplinary Antimicrobial Resistance (HEAT‐AMR) Research Group University of Alberta School of Public Health Edmonton Alberta Canada

**Keywords:** digenetic trematodes, host generalists, host range, host specialists, host specificity

## Abstract

Digenetic trematodes are valuable study organisms for exploring how biodiversity influences disease. In this study, we investigated the relationship between snail richness and trematode infection prevalence using data from a 4‐year study (2019–2022) of eight wetland sites in Alberta, Canada. Trematode species were classified as specialists or generalists at the first‐intermediate host level, and generalized linear mixed‐effects models were employed to assess the relationship between snail richness and overall, generalist, and specialist infection prevalence. The findings indicate that as snail richness increased, there was a significant increase in the overall and generalist infection prevalence. This trend was also noted for specialist infections but was not significant. A notable decline in infection prevalence was observed for all three categories in the final sampling year compared with the first year. Additionally, we found no relationship between snail richness and trematode richness. Trematode and snail–trematode interaction sample completeness and rarefaction analyses indicated that high sample coverage was obtained, but further trematode species remain to be cataloged. We also uncovered interesting one‐off infections that could have important implications for disease monitoring and management strategies that rely on snail hosts, emphasizing the need for continued surveillance of host–parasite relationships.

## Graphical abstract

We find that higher snail richness correlated with increased overall and generalist trematode infections, while specialist infections showed a similar but nonsignificant trend. The study also highlighted potential host‐switching events and the impact of long‐term sampling on parasite communities, emphasizing the need for continued monitoring of host–parasite interactions.

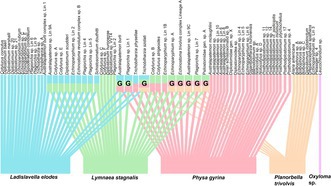

## 1 Introduction

Parasites rely on host organisms to complete their life cycles, making host diversity a critical factor in shaping parasite diversity. The “host diversity begets parasite diversity” hypothesis posits that as host richness increases, so does parasite richness (Hechinger and Lafferty 2005; Kamiya et al. 2014; Wood and Johnson 2016). By increasing and diversifying the available host species in an area, more niches become available for parasites to occupy (Wood and Johnson 2016). In cases of habitat restoration, where host species are returning to the sites, parasite richness is expected to increase along with host diversity (Hechinger and Lafferty 2005). Empirical studies have demonstrated that increasing host richness can increase parasite richness and prevalence (Hechinger and Lafferty 2005; Fredensborg et al. 2006; Hechinger et al. 2007; Thieltges et al. 2011; Kamiya et al. 2014; Johnson et al. 2016). However, the previous studies that have found a positive relationship between host diversity and parasites have primarily measured host species richness and parasite species richness (e.g., Hechinger and Lafferty 2005; Hechinger et al. 2007; Thieltges et al. 2011; Kamiya et al. 2014; Johnson et al. 2016). Understanding these relationships requires consideration of host specificity, as parasite transmission dynamics can vary greatly depending on whether parasites are generalists or specialists.

Digenetic trematodes are ubiquitous parasitic flatworms with complex life cycles involving several hosts, in which mollusks are critical components. Trematodes are often considered more specific to their snail first intermediate hosts than to their vertebrate definitive hosts, presumably due to their long history of coevolution (Adamson and Caira 1994; Esch and Fernandez 1994). Their life cycles begin when trematode eggs are released from the definitive host into the environment. These eggs typically hatch into miracidia, which infect the first intermediate host (usually a gastropod mollusk). Within the molluscan first intermediate host, the trematode will asexually produce free‐living larval stages known as cercariae. The cercariae emerge from the snail and infect either a second intermediate host, where they will encyst as metacercariae, or a definitive host in which they will sexually reproduce to start the life cycle anew (Esch and Fernandez 1994; Galaktionov and Dobrovolskij 2003).

Trematodes at a particular life stage can be host generalists, meaning they can infect many different species of host, or host specialists, infecting a single or a few host species (Adamson and Caira 1994; Poulin and Mouillot 2004, 2005). The most straightforward appraisal of trematode host specificity is the number of host species it can infect, that is, the host range (Rohde 1980; Poulin and Mouillot 2003). The host range of digenetic trematodes is shaped by filters such as encounter and compatibility (Combes 2001; Frankel et al. 2015). Encounter filters include behavior, the habits of the parasites and hosts that allow them to come into contact, and the availability of hosts in the environment (Combes 2001). Compatibility filters encompass resources (physical space and sustentative qualities) and defense (ability of the host immune system to neutralize the parasite) (Combes 2001). Should compatibility not be a factor, trematode infections should be heaviest in the hosts that are most commonly encountered (Frankel et al. 2015; Johnson et al. 2019; Manzoli et al. 2021; Stewart Merrill et al. 2022). Previous research has emphasized the importance of these filters in shaping parasite transmission and host associations. For example, work on trematode infections in amphibians and snails has demonstrated that parasite infection success depends on opportunities for host contact and the susceptibility of host species to infection (Johnson et al. 2008, 2013).

Trematodes can be true generalists that infect many organisms of different families, or generalists that exhibit a preference for one group of hosts while occasionally infecting others opportunistically (Rohde 1980; Poulin and Mouillot 2003; Lane et al. 2015). It has been theorized that specialism arose to increase transmission success. If life cycle stages are released into the environment to align with peak host abundance, this could lead to specialization in one host over time (Adamson and Caira 1994). However, host specialists may be more affected by changes in host composition or their environment, whereas generalists may be more effective at navigating such changes (Manzoli et al. 2021). Within this study, we will define trematodes as host specialists or generalists based on the number of molluscan first intermediate hosts they are observed to infect.

The free‐living larval stages of trematodes can be morphologically similar to one another (Blasco‐Costa et al. 2016), potentially leading to a group of cryptic species being classified as a single species. Cryptic species are morphologically indistinguishable but genetically distinct lineages that were previously classified as a single species (Miura et al. 2005; Bickford et al. 2007). If this “species” was found to infect multiple host species, it would be considered a generalist. However, once the cryptic species are differentiated using genetic sequences, the species complex may be discovered to be several specialist species (Miura et al. 2005; Poulin and Keeney 2008; Lane et al. 2015; Gordy et al. 2017). Cryptic species are common within the Trematoda (Pérez‐Ponce de León and Poulin 2018; Cribb et al. 2025) with evidence of their occurrence in at least 23 families (Cribb et al. 2025). With the implementation of molecular tools in field surveys, we can now discriminate between morphologically indistinct species (Blasco‐Costa et al. 2016).

Snails are integral components of trematode life cycles, and it is possible that losing a snail species from an environment could have a much larger impact on the trematode community composition than removing a single definitive host species (Lafferty et al. 2006). Because snails play such an important role in the life cycles of digenean trematodes, it is important to understand the relationship between snail species richness and trematode infection in a natural ecosystem. We aimed to examine if increasing snail diversity impacted host‐specialist and generalist trematodes differently. Using snail–trematode data from a longitudinal study performed in eight reclaimed wetlands in Alberta, Canada, we examined the relationship between snail host richness and the prevalence of trematode infection using annual sampling data.

## 2 Materials and Methods

### 2.1 Sample Collection

**FIGURE 1:**
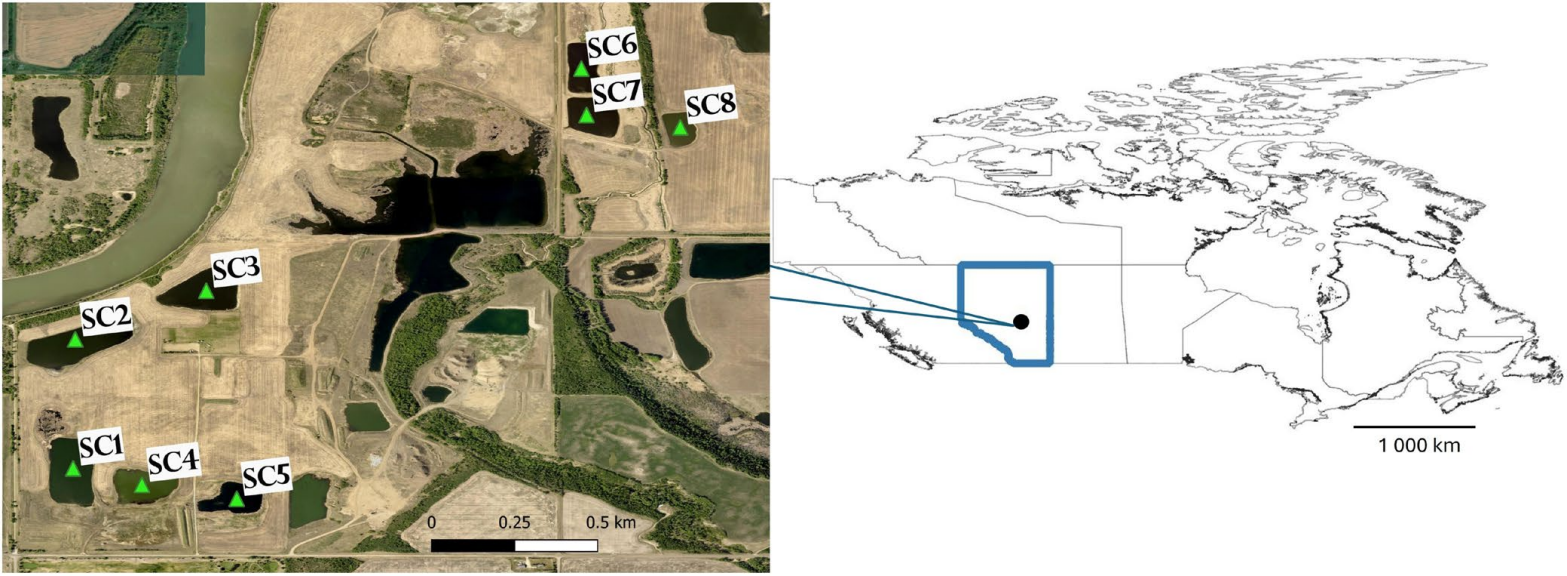
Map of the collection sites located approximately 25 km northeast of Edmonton, Alberta, Canada, in Strathcona County. Each pond is designated by a green triangle. Made using QGIS Development Team (2024) with shapefiles from Statistics Canada (2011). SC, Strathcona County, Alberta.

As part of a larger study, snails were collected during four summers (June to September 2019–2022) in the morning to early afternoon from eight reclaimed wetlands approximately 25 km northeast of Edmonton, Alberta, Canada (Figure 1, Table 1). These sites were chosen based on information provided by an industrial partner that had mined these sites previously. Each site was visited between 5 and 7 collection days per summer due to occasional industrial activity that disrupted access to the sites. Industrial activity in the region could have potentially influenced wetland conditions (e.g., through changes in water quality and shoreline vegetation, or disruption of definitive host use of the sites). We did not directly measure such impacts during this study. Complete survey methods and results are outlined in McPhail et al. (2025).

**TABLE 1:** Location of sampling sites in Alberta, Canada where snails were collected from 2019 to 2022.

Site	Latitude	Longitude	Area (ha)
SC1	53.63254	−113.29271	3.66
SC2	53.63841	−113.29261	3.44
SC3	53.64062	−113.28664	3.84
SC4	53.63179	−113.28957	2.44
SC5	53.63116	−113.28526	1.81
SC6	53.65072	−113.26956	1.79
SC7	53.64861	−113.26933	2.20
SC8	53.64805	−113.26506	1.40

*Note:* The area of each pond is reported in hectares.

Abbreviation: SC, Strathcona County, Alberta.

Briefly, all snail species present were collected at each site by hand, dip net (25 cm diameter), or handheld sieve (22 cm diameter) and transported to the laboratory, where they were shed for trematode cercariae. Snails > 1 cm in length were collected during the timed collection periods where the aim was to collect 200 snails per site per collection day, and no snails were returned to the ponds. Snails were identified morphologically based on descriptions in the *Aquatic Invertebrates of Alberta* (Clifford 1991), and snails were kept in well plates with artificial spring water (Ulmer 1970) under timed fluorescent lights on a 12‐h light–dark cycle (Gordy et al. 2016). After 24 h, the well plates were examined using a stereo microscope to look for cercariae (Gordy et al. 2016). The snails were not dissected to enumerate nonpatent infections, meaning that all infections observed were able to viably produce free‐living larval stages. Snail shedding was used to assess trematode infections, with the recognition that this method may underestimate trematode infection prevalence. This approach is consistent with previously published studies that have characterized snail–trematode interactions using snail shedding (e.g., Gordy et al. 2016; Duan et al. 2021), and was applied consistently across all sites and years.

Cercariae from each snail were preserved in 95% ethanol (Gordy et al. 2016). The goal was to preserve approximately 50 cercariae per sample; however, this depended on the quantity that had emerged from each snail. Cercariae were identified to the family level using the *Handbook of Trematodes of North America North of Mexico* (Schell 1985) and images of trematodes known to be present in central Alberta (Gordy et al. 2016). DNA was extracted from the cercariae samples using a DNEasy Blood and Tissue Kit (Qiagen, Germany), amplified using Polymerase Chain Reaction (PCR), and prepared for Sanger sequencing using a PCR Cleanup kit (Truin Science Ltd., Edmonton, Canada) (Webster 2009; Gordy et al. 2016; McPhail et al. 2021). To identify samples to the species level, the Cytochrome oxidase subunit I (*COI*) was sequenced for most trematodes, with the exception of the Echinostomatidae, for which the Nicotinamide Adenine Dinucleotide dehydrogenase subunit 1 (*nad1*) gene was sequenced (Gordy et al. 2016).

### 2.2 Statistical Methods

#### 2.2.1 Network Metrics

Network metrics of the snails and trematodes collected were assessed using the R (R Core Team 2023) package bipartite (Dormann et al. 2024) in RStudio (Posit Team 2023). A binary snail–trematode interaction matrix for data from all sites pooled was assembled and visualized using the function “plotweb” to assess the specialism of the interactions observed at the study sites. The binary method (a value of 1 if that snail–trematode interaction was observed, and 0 if not, across all study years) was chosen over the quantitative method (a count of each time a snail–trematode relationship was observed across study years) so that we could highlight interesting one‐off infections. We used the “specieslevel” function in the bipartite package (Dormann et al. 2024) to calculate the paired difference index (PDI) value for each species, to determine whether the trematode was a specialist or generalist at the first intermediate host level. Poisot et al. (2011) first put forth this metric to measure specificity. The PDI is calculated as follows: $\mathrm{PDI}=\frac{\Sigma \left(P_{1}-P_{i}\right)}{\left(H-1\right)}$, where *P*_1_ represents the number of interactions in the most common link, *P*_*i*_ refers to the number of interactions in each of the remaining links, and *H* is the number of potential interactors (five snail species in this case) (Dormann et al. 2024). Because a binary interaction matrix was used, *P*_1_ and *P*_*i*_ can only be 1 (if the snail–trematode interaction was present) or 0 (if absent). Further details can be found within the *bipartite* documentation (Dormann et al. 2024). For the analyses, a PDI value of 0 meant a true generalist, and a value of 1 meant a true specialist. The PDI values were visualized with a histogram to determine a cutoff for specialist species (discussed further below).

#### 2.2.2 Sample Completeness

Using the R package iNEXT.4steps (Chao et al. 2020), sampling completeness for trematode species and snail–trematode interactions was assessed. For all analyses, only those trematodes that had been identified to the species level using DNA sequence data were included. The trematode identifications were totaled across all eight sites and four collection years. A count of each time a snail–trematode interaction occurred across all sites and study years was used for the snail–trematode interaction sampling completeness assessment. Interaction completeness is often reported in plant–pollinator networks to estimate the number of interactions occurring in the study area and ensure adequate sampling (e.g., Chacoff et al. 2012; Souza et al. 2018; Worthy et al. 2023). We posited that it could be useful in reporting snail–trematode relationships. Rarefaction analyses were performed for both trematode species and snail–trematode interaction data using the iNEXT package (Hsieh et al. 2016) to provide insight into the adequacy of the sampling effort and the possibility that more species would be added with additional sampling. Rarefaction curves were extrapolated up to an end‐point of 2600 individuals, approximately double the observed trematode infections with DNA sequence data (Chao et al. 2020).

#### 2.2.3 Relationship Between Snail Richness and Infection Prevalence

The package *lme4* (Bates et al. 2024) was used to run multilevel generalized linear mixed effects models (GLMM) using a binomial distribution to assess the relationship between snail richness and (1) overall infection prevalence, (2) generalist infection prevalence, and (3) specialist infection prevalence, as well as annual differences. The snail richness values indicate the richness of snail species collected at that study site in that collection year. The overall infection model used all known snail infections, while the specialist and generalist models used only those infections that had been identified using DNA. The models were tested using the four levels of snail richness (each being the number of snail species identified at that site in the indicated collection year), as well as binning them into levels “low” (snail richness = 2 or 3) and “high” (snail richness = 4 or 5), based on the range of snail richness observed at the study sites. The richness value of 6 was not included because *Aplexa* sp. was only observed on two collection days at one site (SC6), with no patent trematode infections reported. As such, site SC6 was analyzed as snail richness = 5.

The models were compared using the Akaike information criteria (AIC), with lower values indicating a better fit. All models included indicators for year and considered its interaction with snail richness as fixed effects and study site as a random intercept. Individual variables and interactions were assessed for significance using Likelihood Ratio tests (LRTs) with *p* < 0.05 as a cutoff. All models were assessed for goodness of fit by evaluating overdispersion, normality, and homogeneity of residuals. The pairwise contrasts for each of the models were obtained using the package emmeans (Lenth 2024). For both the overall infection model and the generalist infection model, the AIC scores were lower when an interaction between snail richness and year was included, and the interaction term was significant (LRT *p* < 0.05). The assessments of overdispersion, and normality and homogeneity of residuals, for all three models presented did not indicate any concerns with model fit (results not shown). The odds ratios (ORs) represent the relative comparison of the prevalence of infection between compared groups and are presented with 95% confidence intervals (CIs), and were visualized using ggplot2 (Wickham 2016).

#### 2.2.4 Relationship Between Snail Richness and Trematode Richness

We examined the effect of snail richness and year on trematode richness (included as fixed effects) with site included as a random intercept. Using the package glmmTMB (Brooks et al. 2017), we fit a GLMM in R (Posit Team 2023; R Core Team 2023) with a negative binomial distribution to account for overdispersion. Overdispersion was assessed with the performance package (Lüdecke et al. 2021). Likelihood ratio tests were run as described above.

## 3 Results

Across the four study years, 20,557 snails were collected at the eight wetland sites. Six species of snails were observed at the study sites, varying across sites and seasons. These snail species were, in order of abundance: *Lymnaea stagnalis* (9770; 1099 infected), *Physa gyrina* (5435; 331 infected), *Ladislavella elodes* (3210; 253 infected), *Oxyloma* sp. (1096; 3 infected), *Planorbella trivolvis* (848; 100 infected), and *Aplexa* sp. (198) (McPhail et al. 2025). The *Aplexa* sp. snails were only observed and collected on two sampling days (June 27 and July 11, 2022) from a single site (SC6; Figure 1), and no patent (actively shedding) trematode infections were observed. As such, *Aplexa* sp. was omitted from the snail richness values in the models so as not to overstate any relationship between increased snail richness and infection prevalence.

Of the 20,557 snails collected at the eight study sites, 1786 were infected with a digenetic trematode (infection prevalence of 8.69%) (Table 2). Of the trematode samples collected, 1343 returned high‐quality sequences that allowed for identification (75.27%). Seventy‐two species of trematodes were identified, belonging to eight families: Diplostomidae (22 species), Echinostomatidae (15 species), Leucochloridiidae (1 species), Notocotylidae (2 species), Plagiorchiidae (8 species), Psilostomidae (1 species), Schistosomatidae (7 species), and Strigeidae (16 species) (Table S1; McPhail et al. 2025). Seven coinfections were observed during the study: one each in 2019 (site SC7) and 2020 (site SC7), two in 2021 (sites SC4 and SC6), and three in 2022 (one at site SC8 and two at SC4) (McPhail et al. 2025). Each coinfection occurred in a *Lymnaea stagnalis* snail. The two types of cercariae from each snail were preserved separately based on morphological differences. Based on morphological characteristics, six coinfections involved members of Plagiorchiidae and Schistosomatidae, while the remaining included cercariae from Plagiorchiidae and Strigeidae. For four of these coinfections, no high‐quality sequence data were obtained for either cercarial morphotype. For the remaining three coinfections, only one cercarial morphotype from each snail yielded high‐quality sequence data: one *Trichobilharzia szidati*, one *Plagiorchis* sp. Lineage 7, and one *Australapatemon* sp. Lineage 9C. These snails were treated as single infections in the analyses.

**TABLE 2:** Results from 4 years of snail collections at 8 ponds near Edmonton, Alberta, Canada.

Year	Site ID	Snails collected	Infected snails	Infections with species IDs	Snail richness
2019	SC1	768	125	90	4
	SC2	1210	88	79	3
	SC3	627	4	4	3
	SC4	624	160	103	3
	SC5	545	10	5	4
	SC6	1449	85	60	4
	SC7	863	101	83	5
	SC8	733	84	82	3
2020	SC1	744	23	19	3
	SC2	890	91	68	3
	SC3	349	1	0	2
	SC4	195	10	9	2
	SC5	300	11	4	5
	SC6	600	45	35	5
	SC7	590	148	95	5
	SC8	60	15	10	3
2021	SC1	229	25	21	2
	SC2	152	30	24	4
	SC3	1535	109	85	4
	SC4	520	81	59	2
	SC5	145	19	10	3
	SC6	654	69	62	5
	SC7	292	35	33	5
	SC8	20	2	2	3
2022	SC1	434	18	15	4
	SC2	1126	65	49	5
	SC3	1003	67	51	4
	SC4	481	73	55	2
	SC5	125	6	5	5
	SC6	1240	33	24	5
	SC7	615	18	14	4
	SC8	1439	135	88	2

*Note:* The infected snail values are all snails that had trematode cercariae emerging. Infections with species IDs are those identified using genetic sequences. For site locations, see Figure 1.

Abbreviation: SC, Strathcona County, Alberta.

### 3.1 Network Metrics

**FIGURE 2:**
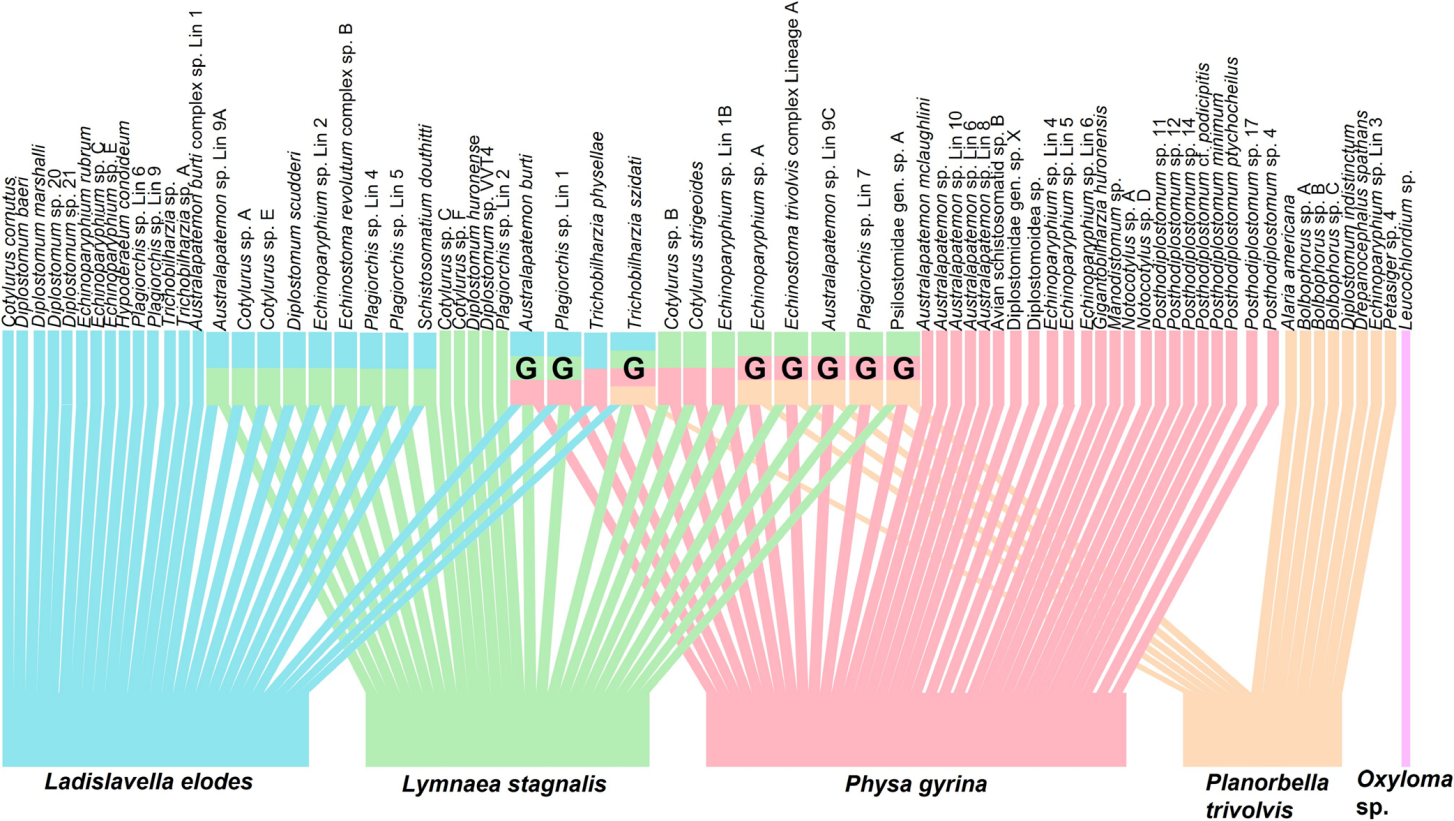
Binary network diagram of snail–trematode relationships observed across all study sites and years. The snail hosts are found along the bottom, and each colored block along the top represents a trematode species. Trematode species names are listed vertically above their corresponding block. “G” indicates a generalist species (infecting 3 or 4 snail species) based on snail‐trematode relationships observed throughout the four sampling years (2019–2022); the remaining trematodes were designated as specialists. Multicolored trematode blocks indicate relationships with multiple snail species, corresponding to the color of the snail blocks along the bottom. Created using the package bipartite (Dormann et al. 2024).

Based on the observed PDI values for the snail–trematode relationships, there was a natural break in the PDI values at 0.6, where species with a PDI > 0.6 were specialists (64 species), and species with a PDI < 0.6 were generalists (8 species) (Table S1). Based on this index, specialist trematodes were those infecting 1 or 2 snail hosts, while generalists were those infecting 3 or 4 snail hosts (Figure 2). The specialist and generalist designations for the trematode species in this study are based on the snail‐trematode relationships observed during 4 years of this study alone, with data from past studies not included. Of the 1343 trematode samples identified using molecular methods, 846 were assigned generalist and 497 were assigned specialist, making an approximate ratio of 1.7 generalist infections for every specialist infection.

### 3.2 Sample Completeness

**FIGURE 3:**
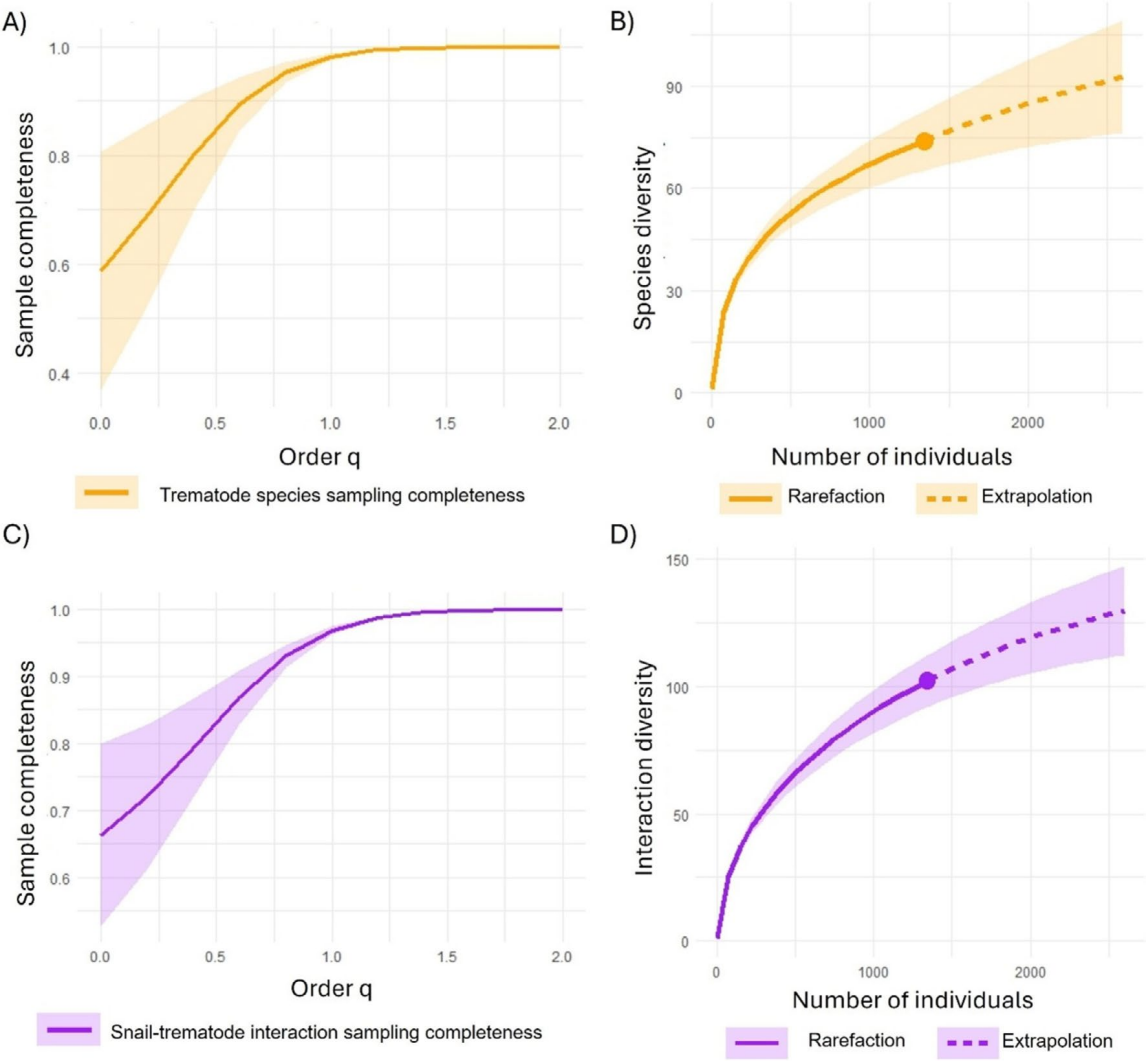
Sample completeness and rarefaction curves for trematode species (orange) and snail‐trematode interactions (purple). The shaded areas indicate the 95% confidence interval. For the sample completeness profiles (A and C), the levels of q indicate: Sampling completeness of dominant trematode species or snail–trematode interactions (*q* = 2), typical trematode species or snail–trematode interactions (*q* = 1), and trematode species or snail–trematode interaction richness (*q* = 0). For the rarefaction curves (B and D), the solid line indicates the interpolated values, and the dashed lines indicate the rarefaction extrapolation. Data used are either trematodes collected or snail–trematode interactions from all eight wetlands from 2019 to 2022. (A) Sample completeness profile for all trematode species. (B) Rarefaction curve illustrating trematode species richness. (C) Sample completeness profile for all trematode–snail interactions. (D) Rarefaction curve illustrating snail–trematode interaction diversity.

The results of the trematode species sampling completeness assessment indicated that we captured 100% of the dominant (highly frequent) species (*q* = 2), 98% of the typical (frequent) species (*q* = 1), and 58% of the diversity of all species (*q* = 0) (Figure 3A). The snail–trematode interaction sampling completeness assessment showed that we detected 100% of the dominant interactions (*q* = 2), 97% of the typical interactions (*q* = 1), and 66% of the diversity of all interactions (*q* = 0) (Figure 3C).

### 3.3 Relationship Between Snail Richness and Infection Prevalence

**FIGURE 4:**
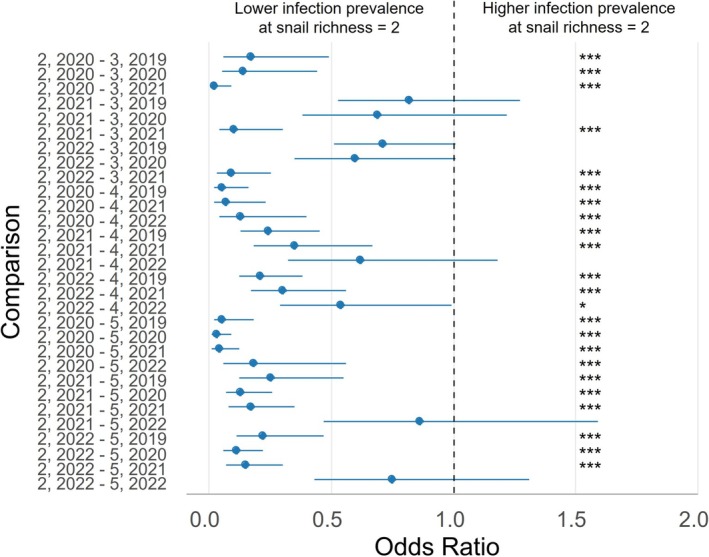
Odds ratios (ORs) with 95% confidence intervals (CIs) from pairwise comparisons of snail richness levels across study years for the overall infection model. ORs < 1.0 indicate lower infection prevalence at snail richness = 2. Comparisons are labeled as “2, year – snail richness, year,” where “2, year” is the reference. Data include all snails infected with a digenetic trematode within the indicated year. Some pairwise comparisons were nonestimable due to the absence of sites with a given richness in that year (See Figure S2 for nonestimable comparisons). *p < 0.05; **p < 0.01; ***p ≤ 0.0001.

**FIGURE 5:**
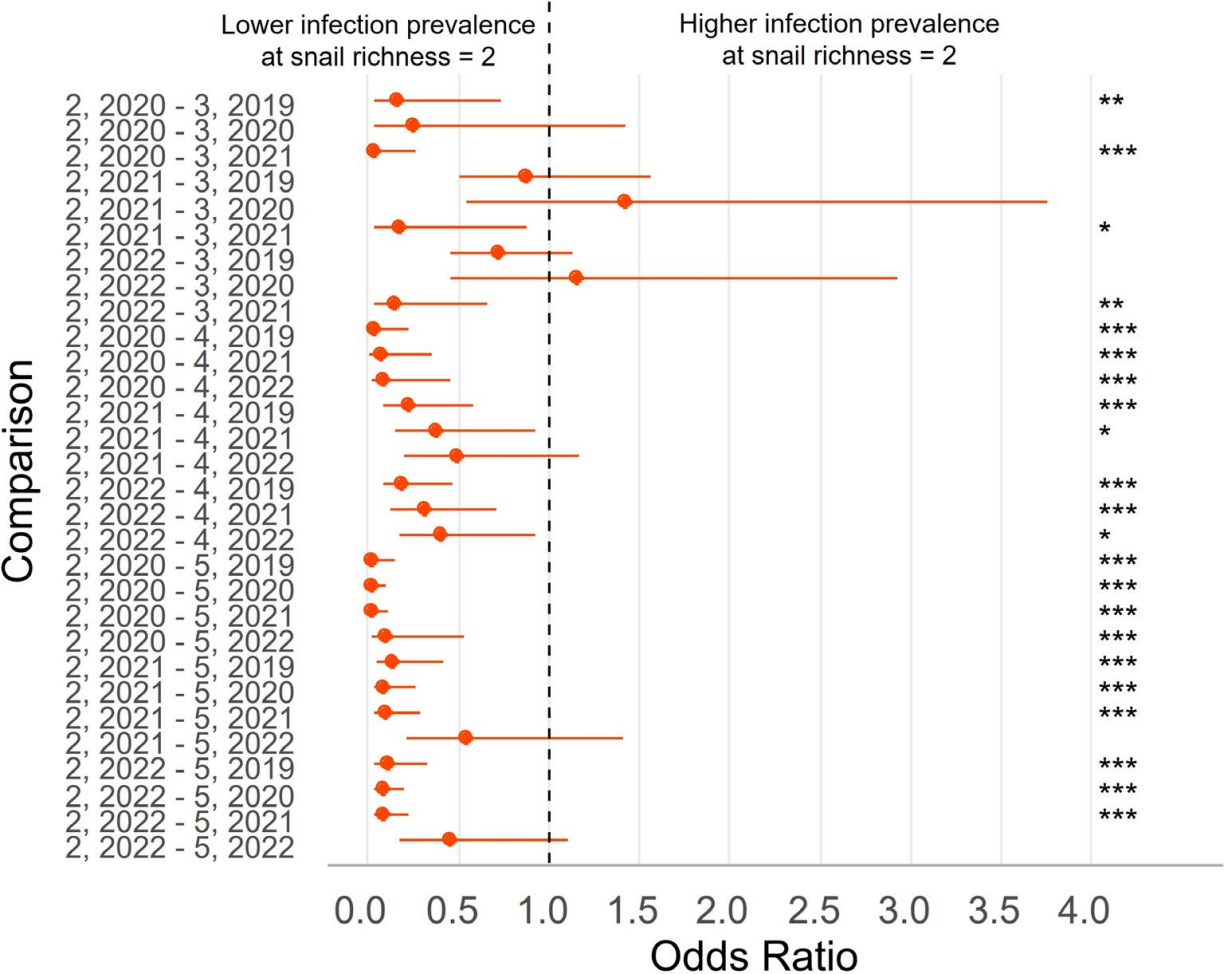
Odds ratios (ORs) with 95% confidence intervals (CIs) from pairwise comparisons of snail richness levels across study years for the generalist infection model. ORs < 1.0 indicate lower infection prevalence at snail richness = 2. Comparisons are labeled as “2, year – snail richness, year,” where “2, year” is the reference. Data include the trematode infections that were identified using DNA sequencing and determined to be generalist trematodes based on the paired differences index (PDI) calculated using the bipartite package (Dormann et al. 2024), see section 2.21 for more details. Some pairwise comparisons were nonestimable due to the absence of sites with a given richness in that year (See Figure S3 for nonestimable comparisons). *p < 0.05; **p < 0.01; ***p ≤ 0.0001.

The models using the four snail richness levels had lower AIC scores than those binning snail richness into “low” and “high”. For the 32 site and year combinations, the snail richness varied between two and five species. The proportion of each snail richness level is presented in Table 3 (pooled), Figure S1 (by year), and information on snail species observed within each snail richness level and site is presented in Table S2. We evaluated the relationship between snail richness, year, and infection prevalence across three models: overall, generalist, and specialist infections. In the models for overall and generalist infections, LRTs indicated significant effects of snail richness (*p* < 0.001), year (*p* < 0.001), and their interaction (*p* < 0.001) (Tables S3 and S4). Pairwise contrasts compared snail richness level 2 to higher levels for all years with available data (Figures 4 and 5). In both models, infection prevalence was generally lower at richness level 2 and increased with snail richness (Figures 4 and 5). For instance, prevalence at level 2 was significantly lower than at level 5 in nearly all estimable comparisons, with only two exceptions in 2022 for each model (Figures 4 and 5, Figures S2C and S3C). The same trend held when comparing level 2 to level 4, with all but one estimable contrast showing significantly lower prevalence at level 2 for each model (Figures 4 and 5, Figures S2B and S3B). Comparisons between levels 2 and 3 also revealed significantly lower prevalence at level 2 in 2021, while the values for 2019 and 2020 showed lower prevalence, but not all were significant (Figures 4 and 5, Figures S2A and S3A).

**TABLE 3:** Collection data categorized by snail richness level.

Snail richness	Snails collected	Overall infections (%)	Generalist infections (%)	Specialist infections (%)
2	3213	325 (10.12%)	182 (5.66%)	50 (1.56%)
3	5053	486 (9.62%)	191 (3.78%)	186 (3.68%)
4	6501	462 (7.12%)	240 (3.69%)	104 (1.60%)
5	5790	513 (9.17%)	233 (4.17%)	157 (2.81%)

*Note:* Overall infections, generalist infections, and specialist infection counts are followed by their prevalence (%), calculated by dividing the count for each by the snails collected within the snail richness level.

The prevalence of the eight generalist trematode species varied across different levels of snail richness (Tables S5 and S6). Three generalist species (*Australapatemon* sp. Lineage 9C, *Echinoparyphium* sp. A, and *Trichobilharzia szidati*) have higher mean prevalence at sites with higher snail richness (richness 4 and 5) (Table S6). *Plagiorchis* sp. Lineage 7 is the largest contributor to generalist infections overall (31.2% of identified generalist infections; Table S5), but its mean prevalence is higher in the lower‐richness sites (Table S6).

**FIGURE 6:**
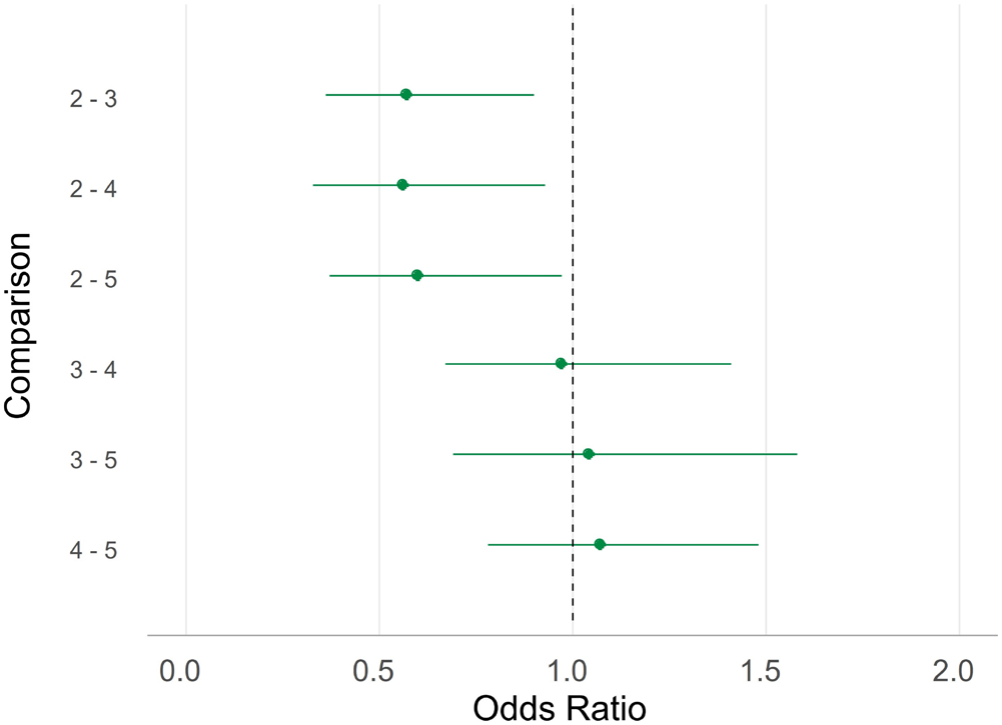
Odds ratios (ORs) with 95% confidence intervals (CIs) from pairwise comparisons of snail richness levels across study years for the specialist infection model. ORs < 1.0 indicate lower infection prevalence at the reference snail richness. Comparisons are labeled as “snail richness, year – snail richness, year,” where the first snail richness is the reference. Data include the trematode infections that were identified using DNA sequencing and determined to be specialist trematodes based on the paired differences index (PDI) calculated using the bipartite package (Dormann et al. 2024); see section 2.21 for more details. This plot is presented to visualize trends only; *p* values are not reported because snail richness was not significant in the specialist model (see Table S7).

The final model examined the relationship between snail richness and the prevalence of specialist infections. This model did not include an interaction, as the AIC score was lower without it, and the interaction was not significant when comparing the models using an LRT. Year had a significant effect (*p* < 0.001), but snail richness was not significant overall (*p* = 0.09; Table S7). We opted to include it to determine if there was an association between snail richness and specialist infection prevalence. Unlike the previous two models, all pairwise contrasts were estimable. Specialist infection prevalence was generally lower at richness level 2 compared to higher levels (Figure 6, Figure S4), though ORs among higher richness levels (e.g., 3 vs. 4 or 5) were close to 1.0. While these results suggest a trend toward increasing specialist infection with greater snail richness, the effect was not statistically significant.

**FIGURE 7:**
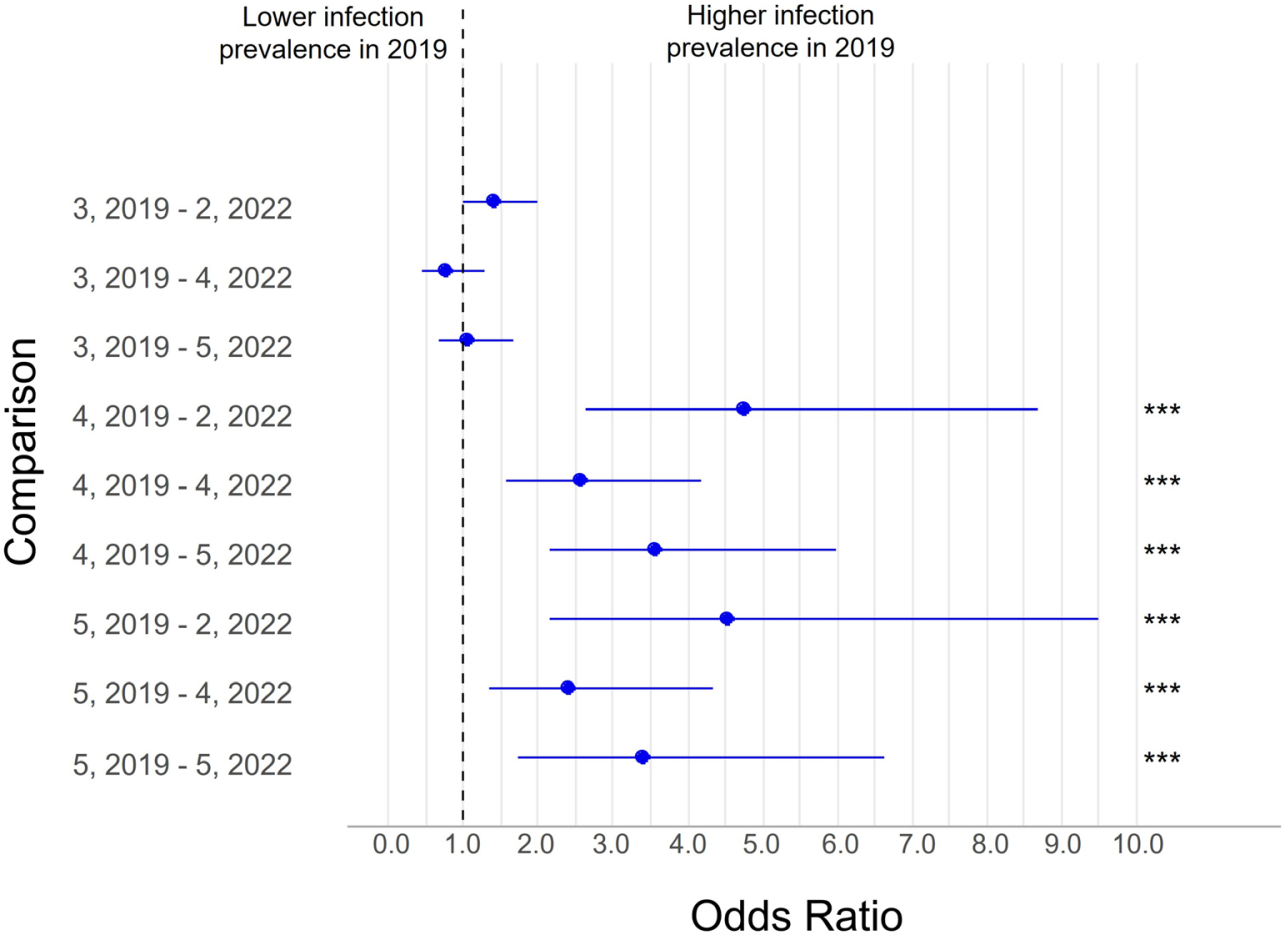
Odds ratios (ORs) with 95% confidence intervals (CIs) for trematode infection prevalence at each snail richness level for the year 2022 (last year of snail sampling) compared to 2019 (first year of snail sampling) using the overall infection model. Data include all snails infected with a trematode in 2019 and 2022. ORs > 1.0 indicate that the prevalence of infection with a digenetic trematode was greater in 2019 than in 2022 at the indicated snail richness level. Comparisons are labeled as “snail richness, year – snail richness, year,” where the first “snail richness, year” is the reference. Some pairwise comparisons were nonestimable due to the absence of sites with a given richness in that year (See Figure S5 for nonestimable comparisons). *p < 0.05; **p < 0.01; ***p ≤ 0.0001.

To understand if there was an effect of year on trematode infection prevalence, ORs were compared between the first sampling year (2019) and the last sampling year (2022) across all snail richness levels (Figure 7) using the pairwise contrasts from the overall infection model. In 2019, the prevalence of infection was significantly higher for richness levels 4 and 5 when compared to 2022 (Figure 7, Figure S5). The prevalence could not be calculated for snail richness = 3 in 2022 because no pond exhibited a snail richness of 3 in 2022 (Figure S5). Similar trends were noted when analyzing the pairwise contrasts from the generalist (Figure S6) and specialist (Figure S7) infection models.

### 3.4 Relationship Between Snail Richness and Trematode Richness

The likelihood ratio tests determined that snail richness (*p* = 0.45) and year (*p* = 0.13) were not significant to the model. As such, we did not continue modeling this relationship.

## 4 Discussion

This study examined the relationship between snail richness and overall infection prevalence, generalist infection prevalence, and specialist infection prevalence using data from 4 years of snail–trematode collections. These collections revealed five snail species that were hosting 72 trematode species in a relatively small geographic area. We found that as snail richness increased, so did the prevalence of trematode infection when evaluating overall infection prevalence, infection with generalists, and infection with specialists, though this trend was not significant for the specialist trematodes. Notably, there was no relationship between snail richness and trematode richness. We also observed a decrease in infection prevalence in the fourth year of collection (2022). These results indicate that although infection prevalence tended to increase with snail richness, trematode species richness did not show a similar pattern.

Increased infection prevalence with increased snail richness was significant for both overall infection and generalist infections. Specialist infections (*n* = 497) were observed less frequently than generalist infections (*n* = 846) throughout the study. Additionally, generalist trematodes are likely to be more robust in the face of host community composition changes due to their ability to infect several hosts (Manzoli et al. 2021). These results are interesting in the context of the relationship between biodiversity and disease. While this relationship differs from the pattern reported under the “diversity begets diversity” hypothesis (Hechinger and Lafferty 2005; Kamiya et al. 2014), which typically emphasizes a positive association between host diversity and parasite richness, our findings instead highlight a positive relationship between host richness and infection prevalence. This suggests that increased host diversity may not only support increased richness as observed in previous studies (Hechinger and Lafferty 2005; Kamiya et al. 2014) but may also enhance opportunities for transmission, especially if multiple host species are competent. It is possible that this relationship could plateau as more snail species are added, meaning that infection prevalence would not continue increasing as additional host species are added. A previous analysis of 205 biodiversity–disease relationships found that amplification effects can level off, resulting in an asymptotic relationship between biodiversity and disease (Halliday and Rohr 2019). These findings emphasize the importance of studying biodiversity–disease dynamics within ecologically relevant and complex scenarios.

The relationship between increased snail richness and trematode prevalence is driven by the eight generalist trematode species that account for 846 of the 1343 infections. We did not find evidence that the increase in trematode infection prevalence with snail richness was driven by a positive relationship between snail and trematode richness. Instead, certain generalist species exhibited elevated prevalence in snail communities with higher richness (4 and 5 species), and two trematode species (*Plagiorchis* sp. Lineage 7 and *T. szidati*) accounted for a large proportion of all identified trematode species. One explanation is that increased encounter rates and host availability in diverse snail communities benefited generalist species (Manzoli et al. 2021). Additionally, the generalist species were compatible with more snail species, allowing for infection prevalence to increase with snail richness.

It was unsurprising that there was no evident relationship between snail richness, year, and trematode richness, even though we observed a positive relationship between snail richness and trematode infection prevalence. A handful of snail species are hosting 72 trematode species at the eight wetland sites, as such snail species are susceptible to infection by multiple trematode species. Past research that has found a positive relationship between host richness and trematode richness has investigated definitive host diversity (e.g., Hechinger and Lafferty 2005; Thieltges et al. 2011). A previous study that examined the effect of first intermediate host richness on trematode richness across 25 biogeographic regions in Europe did not find a significant relationship for both autogenic (those that complete their life cycles within freshwater habitats) and allogenic (those that complete their life cycles outside of freshwater habitats) species (Esch et al. 1988; Thieltges et al. 2011). They did, however, uncover strong relationships between definitive hosts and parasite richness for autogenic and allogenic parasites and between latitude and definitive host richness (Thieltges et al. 2011). In this study, host richness and trematode richness were compiled from information reported in the *Limnofauna europaea* (Illies 1978; Thieltges et al. 2011), and trematode species without known definitive hosts were excluded from the analyses. A meta‐analysis of 38 studies focusing on the relationship between the species richness of metazoan and protozoan parasites and their hosts found a strong positive correlation (Kamiya et al. 2014), and snails were the only host group not significantly related to parasite richness (Thieltges et al. 2011; Kamiya et al. 2014). These findings suggest that while host diversity often promotes parasite diversity, this pattern may not occur for all host groups.

For all three models presented, there was a lower prevalence of infection in 2022 compared with the reference year of 2019, and this was significant for both the overall and generalist infection models. In the first 3 years of the study, the trematode infection prevalence was between 9% and 10.5%. However, in 2022 the infection prevalence was only 6.4%. Additionally, snail richness fluctuated between years at the study sites. It is possible that by removing infected snails for 4 years, trematode larval stages were also removed from the environment. These may have otherwise been shed and could have continued the life cycle by infecting the next host. Alternatively, it is possible that snail abundance varied between years due to the impact of trematode infections decreasing snail fecundity (Lafferty 1993; Fredensborg et al. 2005), or that environmental stressors impacted snails present in the ponds.

Such environmental stressors could include cyanobacteria blooms, increasing temperatures, and/or exposure to pollutants. Snail species may be affected differently by environmental stressors such as cyanobacterial blooms (Lance et al. 2010), which occurred throughout the study (B. McPhail, personal observation) and can cause a decrease in snail abundance (Gérard et al. 2008; Lance et al. 2010; Gérard and Lance 2019) and fecundity (Gérard et al. 2005, 2008; Lance et al. 2007). Additionally, there is evidence that as water temperatures increase, snails grow larger (Paull and Johnson 2011; Moore et al. 2021) and can exhibit increased fecundity up to an optimal temperature (Paull and Johnson 2011), after which the percent of viable eggs produced and the total eggs produced decreases (Moore et al. 2021). Larger snails have also been shown to exhibit higher trematode infection prevalence (Kuris and Lafferty 1994; Jokela and Lively 1995; Miura and Chiba 2007; Tolley‐Jordan and Chadwick 2019). Average maximum air temperatures in June, July, and August were higher in 2021 than in 2019 and 2020, while 2022 was similar to 2021 in July and August (Table S8; Environment and Climate Change Canada 2025). These warmer conditions may have impacted water temperatures. Furthermore, exposure to a mixture of 14 common environmental pollutants was shown to negatively affect the survival, growth, shell length, hatch success, snail mass, and total eggs produced of *L. stagnalis* even at low levels (0.05 μg/L) (Moore et al. 2021). Should a molluscan potential host be affected by an environmental stressor, this could alter the susceptibility to trematode infection (Lafferty 1997; McDowell et al. 1999; Paull and Johnson 2011). Evidently, these ponds are dynamic ecosystems that could be influenced by multiple factors that affect snail species composition and infection dynamics.

Capturing both common and rare snail–trematode interactions is essential for understanding the full complexity of trematode communities and their transmission dynamics. The sampling completeness assessments for the trematode and snail–trematode interaction data indicated that the dominant species were captured entirely, and the typical species were nearly completely captured. However, there remain species to be found, which was confirmed through the rarefaction curves and the species richness level (*q* = 0) of the sampling completeness analyses (Figure 3A–D). These analyses were run using data from this study alone. We opted for a binary network matrix to study the snail–trematode relationships observed in this study because several rare and unusual snail–trematode combinations were down‐weighted when using a quantitative matrix and were not as evident in the quantitative network diagram (Figure S8). For some trematode species, there was a common snail–trematode interaction observed hundreds of times alongside rare one‐off infections. For example, the avian schistosome species *Trichobilharzia szidati* is known to infect the great pond snail *Lymnaea stagnalis* (Brant and Loker 2009; Gordy et al. 2018; Rudko et al. 2019; Skála et al. 2020). We observed this relationship 190 times in 4 years and confirmed the observations using genetic sequences. However, we also found it to be infecting *Physa gyrina* (*n* = 2; August 2020 and July 2022), *Planorbella trivolvis* (*n* = 1; July 2019), and *Ladislavella elodes* (*n* = 1; August 2019). To our knowledge, *T. szidati* has not been reported to infect these three snail species previously. Because cercariae were not pooled between samples but collected directly from the shedding snail and preserved in a corresponding labeled tube, we are confident that these identifications were not made in error. It is currently unknown how these one‐off infections occur because the snails were collected from the natural environment, but possible explanations could include environmental stressors previously discussed above or potential preconditioning of the snails by prior infections. An example of this was demonstrated in a *Schistosoma mansoni‐*resistant strain of *Biomphalaria glabrata* (strain 10‐R2; Richards 1973; Kassim and Richards 1979; Lie et al. 1977b), which became susceptible to *S. mansoni* infection after first being infected with echinostome species (*Echinostoma paraensei*, *E. lindodense*, *E. liei*) (Lie et al. 1977a, 1977b; Loker et al. 1986). This highlights the importance of revisiting and questioning long‐held assumptions about host specificity in trematode–snail relationships.

As digenetic trematode researchers, we often make assumptions regarding the specificity of the relationship between the first intermediate snail hosts and their trematode parasites. To our knowledge, since the discoveries made by Cort in Michigan nearly 100 years ago (Cort 1928; Cort and Brooks 1928), no records of *Planorbella* spp. snails as hosts for avian schistosome trematodes have been published. In 2015, a single *Pl. trivolvis* snail was found to be infected with an avian schistosome (Avian schistosomatid sp. C) in central Alberta (Gordy et al. 2018), as part of a larger snail–trematode study (Gordy et al. 2016; Gordy and Hanington 2019). Avian schistosomatid sp. C was then included among the avian schistosomes tracked using quantitative polymerase chain reaction (qPCR) during a longitudinal study of Michigan lakes (McPhail et al. 2021; Rudko et al. 2022). This species was found to be an important contributor to the avian schistosome community in northern Michigan lakes (Rudko et al. 2022) and was the most abundant and widespread avian schistosome in southern Michigan lakes (Soper et al. 2023). The contribution of Avian schistosomatid sp. C would have gone unnoticed if we had not investigated *Pl. trivolvis* snails. In the case of dangerous infectious agents like *Schistosoma mansoni* , even a few one‐off infections that allow for the release of cercariae can have far‐reaching consequences that cannot be monitored and mitigated if they go unnoticed.

In conclusion, we aimed to examine the relationship between snail richness and overall infection prevalence, as well as the infection prevalence of generalist and specialist trematodes. Both the trematodes and snail–trematode interactions were well sampled for the dominant and typical species and interactions, but less so for overall species and interaction richness. Our findings demonstrate that the prevalence of infection with a digenetic trematode increased as snail richness increased, and this was true for overall infections and generalist trematodes. Additionally, we saw a decrease in infection prevalence in the fourth year of the study, but did not observe a relationship between snail richness and trematode richness. This study also revealed interesting one‐off infections throughout 4 years of snail collections. Future research should focus on determining if the relationship between snail richness and trematode infection prevalence continues to increase in communities with more snail diversity, as this could enhance our understanding of snail–trematode dynamics.

## Author Contributions

**Brooke A. McPhail:** conceptualization (lead), data curation (lead), formal analysis (lead), methodology (lead), writing – original draft (lead). **Carol M. Frost:** formal analysis (supporting), methodology (supporting), writing – original draft (supporting). **Simon J. G. Otto:** formal analysis (supporting), methodology (supporting), writing – original draft (supporting). **Patrick C. Hanington:** conceptualization (equal), funding acquisition (lead), project administration (equal), resources (lead), supervision (lead), writing – original draft (supporting).

## Conflicts of Interest

The authors declare no conflicts of interest.

## Supporting information


**Data S1:** ece372381‐sup‐0001‐supinfo.pdf.

## Data Availability

Scripts and associated data are available on Dryad: https://doi.org/10.5061/dryad.ghx3ffc0v.
