# Supplementary material for: Trematode Infection Prevalence Increases With Snail Richness: Observations From a 4‐Year Study of Snail–Trematode Interactions
Source: Ecol Evol. 2025 Oct 22;15(10):e72381. doi: 10.1002/ece3.72381 (PMC12541670; doi:10.1002/ece3.72381)
Supplement: Supplementary file 1 — Data S1: ece372381‐sup‐0001‐supinfo.pdf. [file ECE3-15-e72381-s001.pdf]

Supporting Information for:

Trematode infection prevalence increases with snail richness: Observations from a four-year study of snail-trematode interactions

Brooke A. McPhail, Carol M. Frost, Simon J. G. Otto, Patrick C. Hanington

Table of Contents:

**Table S1**	Page 2
**Table S2**	Page 5
**Table S3**	Page 8
**Table S4**	Page 9
**Table S5**	Page 10
**Table S6**	Page 12
**Table S7**	Page 13
**Table S8**	Page 14
**Figure S1**	Page 15
**Figure S2**	Page 16
**Figure S3**	Page 17
**Figure S4**	Page 18
**Figure S5**	Page 19
**Figure S6**	Page 20
**Figure S7**	Page 21
**Figure S8**	Page 22
**Literature Cited**	Page 23

Table S1. Paired differences index values for each trematode species in the bipartite binary network matrix. PDI values are followed by the specialist or generalist designation of the species in parentheses. Only snail-trematode relationships observed during the four collection years at the wetland sites are included. Abbreviations: PDI = Paired differences index, G = Generalist, S = Specialist. Snail species: *L. stagnalis* = *Lymnaea stagnalis*; *La. elodes* = *Ladislavella elodes*; *P. gyrina* = *Physa gyrina*; *Pl. trivolvis* = *Planorbella trivolvis.*

Family	Species	PDI (G/S)	Snail host(s)
Diplostomidae	*Alaria americana*	1 (S)	*Pl. trivolvis*
	Bolbophorus sp. A	1 (S)	*Pl. trivolvis*
	Bolbophorus sp. B	1 (S)	*Pl. trivolvis*
	Bolbophorus sp. C	1 (S)	*Pl. trivolvis*
	Diplostomidae gen. sp. X	1 (S)	*P. gyrina*
	Diplostomoidea sp.	1 (S)	*P. gyrina*
	Diplostomum baeri	1 (S)	*La. elodes*
	Diplostomum huronense	1 (S)	*L. stagnalis*
	Diplostomum indistinctum	1 (S)	*Pl. trivolvis*
	Diplostomum marshalli	1 (S)	*La. elodes*
	Diplostomum scudderi	0.75 (S)	*L. stagnalis, La. elodes*
	Diplostomum sp. 20	1 (S)	*La. elodes*
	Diplostomum sp. 21	1 (S)	*La. elodes*
	Diplostomum sp. VVT4	1 (S)	*L. stagnalis*
	Posthodiplostomum cf. *podicipitis*	1 (S)	*P. gyrina*
	Posthodiplostomum minimum	1 (S)	*P. gyrina*
	Posthodiplostomum ptychocheilus	1 (S)	*P. gyrina*
	Posthodiplostomum sp. 4	1 (S)	*P. gyrina*
	Posthodiplostomum sp. 11	1 (S)	*P. gyrina*
	Posthodiplostomum sp. 12	1 (S)	*P. gyrina*
	Posthodiplostomum sp. 14	1 (S)	*P. gyrina*
	Posthodiplostomum sp. 17	1 (S)	*P. gyrina*
Echinostomatidae	*Drepanocephalus spathans*	1 (S)	*Pl. trivolvis*
	Echinoparyphium rubrum	1 (S)	*La. elodes*
	Echinoparyphium sp. A	0.5 (G)	*L. stagnalis, P. gyrina, Pl. trivolvis*
	Echinoparyphium sp. C	1 (S)	*La. elodes*
	Echinoparyphium sp. E	1 (S)	*La. elodes*
	Echinoparyphium sp. Lineage 1B	0.75 (S)	*L. stagnalis, P. gyrina*
	Echinoparyphium sp. Lineage 2	0.75 (S)	*L. stagnalis, La. elodes*
	Echinoparyphium sp. Lineage 3	1 (S)	*Pl. trivolvis*
	Echinoparyphium sp. Lineage 4	1 (S)	*P. gyrina*
	Echinoparyphium sp. Lineage 5	1 (S)	*P. gyrina*

Continued on next page

Table S1 – *Continued from previous page*

Family	Species	PDI (G/S)	Snail host(s)
Echinostomatidae (continued)	*Echinoparyphium* sp. Lineage 6	1 (S)	*P. gyrina*
	Echinostoma revolutum complex sp. B	0.75 (S)	*L. stagnalis, La. elodes*
	Echinostoma trivolvis complex Lineage A	0.5 (G)	*L. stagnalis, P. gyrina, Pl. trivolvis*
	Hypoderaeum conoideum	1 (S)	*La. elodes*
	Petasiger sp. 4	1 (S)	*Pl. trivolvis*
Leucochloridiidae	*Leucochloridium* sp.	1 (S)	*Oxyloma* sp.
Notocotylidae	*Notocotylus* sp. A	1 (S)	*P. gyrina*
	Notocotylus sp. D	1 (S)	*P. gyrina*
Plagiorchiidae	*Manodistomum* sp.	1 (S)	*P. gyrina*
	Plagiorchis sp. Lineage 1	0.5 (G)	*L. stagnalis, La. elodes, P. gyrina*
	Plagiorchis sp. Lineage 2	1 (S)	*L. stagnalis*
	Plagiorchis sp. Lineage 4	0.75 (S)	*L. stagnalis, La. elodes*
	Plagiorchis sp. Lineage 5	0.75 (S)	*L. stagnalis, La. elodes*
	Plagiorchis sp. Lineage 6	1 (S)	*La. elodes*
	Plagiorchis sp. Lineage 7	0.5 (G)	*L. stagnalis, P. gyrina, Pl. trivolvis*
	Plagiorchis sp. Lineage 9	1 (S)	*La. elodes*
Psilostomidae	Psilostomidae gen. sp. A	0.5 (G)	*L. stagnalis, P. gyrina, Pl. trivolvis*
Schistosomatidae	Avian schistosomatid sp. B	1 (S)	*P. gyrina*
	Gigantobilharzia huronensis	1 (S)	*P. gyrina*
	Schistosomatium douthitti	0.75 (S)	*L. stagnalis, La. elodes*
	Trichobilharzia physellae	0.75 (S)	*La. elodes, P. gyrina*
	Trichobilharzia sp.	1 (S)	*La. elodes*
	Trichobilharzia sp. A	1 (S)	*La. elodes*
	Trichobilharzia szidati	0.25 (G)	*L. stagnalis, La. elodes, P. gyrina, Pl. trivolvis*

Continued on next page

Table S1 – *Continued from previous page*

Family	Species	PDI (G/S)	Snail host(s)
Strigeidae	*Australapatemon burti*	0.5 (G)	*L. stagnalis, La. elodes, P. gyrina*
	Australapatemon burti complex sp. Lineage 1	1 (S)	*La. elodes*
	Australapatemon mclaughlini	1 (S)	*P. gyrina*
	Australapatemon sp.	1 (S)	*P. gyrina*
	Australapatemon sp. Lineage 6	1 (S)	*L. stagnalis, P. gyrina*
	Australapatemon sp. Lineage 8	1 (S)	*P. gyrina*
	Australapatemon sp. Lineage 9A	0.75 (S)	*L. stagnalis, La. elodes*
	Australapatemon sp. Lineage 9C	0.5 (G)	*L. stagnalis, P. gyrina, Pl. trivolvis*
	Australapatemon sp. Lineage 10	1 (S)	*P. gyrina*
	Cotylurus cornutus	1 (S)	*La. elodes*
	Cotylurus sp. A	0.75 (S)	*L. stagnalis, La. elodes*
	Cotylurus sp. B	0.75 (S)	*L. stagnalis, P. gyrina*
	Cotylurus sp. C	1 (S)	*L. stagnalis*
	Cotylurus sp. E	0.75 (S)	*L. stagnalis, La. elodes*
	Cotylurus sp. F	1 (S)	*L. stagnalis*
	Cotylurus strigeoides	0.75 (S)	*P. gyrina*

Table S2. Sites included within each snail richness level, along with the abundance of each snail species collected, and the abundance of overall, generalist, and specialist trematode infections. The total infections column also includes snails with trematode infections that were not identified using DNA. *L. stagnalis* = *Lymnaea stagnalis*, *La. elodes* = *Ladislavella elodes*, *P. gyrina* = *Physa gyrina*, and *Pl. trivolvis* = *Planorbella trivolvis*.

Snail richness	Site / Year	Snails collected		Generalist infections	Specialist infections	Total infections
2	SC1 / 2021	*L. stagnalis*	138	9	5	17
		P. gyrina	91	1	6	8
	SC3 / 2020	*L. stagnalis*	29	0	0	1
		Oxyloma sp.	320	0	0	0
	SC4 / 2020	*L. stagnalis*	36	5	0	5
		P. gyrina	159	0	4	5
	SC4 / 2021	*L. stagnalis*	450	48	7	75
		P. gyrina	70	0	4	6
	SC4 / 2022	*L. stagnalis*	411	43	2	59
		P. gyrina	70	2	8	14
	SC8 / 2022	*L. stagnalis*	1436	74	14	135
		P. gyrina	3	0	0	0
3	SC1 / 2020	*L. stagnalis*	139	0	3	5
		P. gyrina	602	6	10	18
		Oxyloma sp.	3	0	0	0
	SC2 / 2019	*L. stagnalis*	10	1	2	4
		La. elodes	1182	22	54	84
		P. gyrina	18	0	0	0
	SC2 / 2020	*L. stagnalis*	148	1	4	5
		La. elodes	427	13	42	76
		P. gyrina	315	2	6	10
	SC3 / 2019	*L. stagnalis*	3	1	0	1
		La. elodes	6	0	0	0
		Oxyloma sp.	618	0	3	3
	SC4 / 2019	*L. stagnalis*	307	60	1	84
		La. elodes	16	0	0	0
		P. gyrina	301	12	30	76
	SC5 / 2021	*L. stagnalis*	136	5	4	18
		P. gyrina	3	0	0	0
		Pl. trivolvis	6	0	1	1
	SC8 / 2019	*L. stagnalis*	178	43	3	46
		P. gyrina	37	1	0	1
		Pl. trivolvis	518	20	15	37
	SC8 / 2020	*L. stagnalis*	15	0	0	1
		P. gyrina	43	2	8	14
		Pl. trivolvis	2	0	0	0

Continued on next page

Table S2 – *Continued from previous page*

Snail richness	Site / Year	Snails collected		Generalist infections	Specialist infections	Total infections
3 (continued)	SC8 / 2021	*L. stagnalis*	17	2	0	2
		Pl. trivolvis	1	0	0	0
		Oxyloma sp.	2	0	0	0
4	SC1 / 2019	*L. stagnalis*	635	75	13	122
		La. elodes	1	0	0	0
		P. gyrina	128	1	1	3
		Pl. trivolvis	4	0	0	0
	SC1 / 2022	*L. stagnalis*	344	7	4	14
		La. elodes	2	0	0	0
		P. gyrina	84	3	1	4
		Pl. trivolvis	4	0	0	0
	SC2 / 2021	*L. stagnalis*	35	1	2	4
		La. elodes	23	0	6	9
		P. gyrina	92	2	13	17
		Pl. trivolvis	2	0	0	0
	SC3 / 2021	*L. stagnalis*	1263	77	2	101
		P. gyrina	266	0	5	7
		Pl. trivolvis	2	0	1	1
		Oxyloma sp.	4	0	0	0
	SC3 / 2022	*L. stagnalis*	798	36	1	46
		P. gyrina	190	12	2	21
		Pl. trivolvis	10	0	0	0
		Oxyloma sp.	5	0	0	0
	SC5 / 2019	*L. stagnalis*	28	0	0	0
		La. elodes	2	0	0	0
		P. gyrina	474	1	4	10
		Pl. trivolvis	41	0	0	0
	SC6 / 2019	*L. stagnalis*	36	1	4	6
		La. elodes	815	6	32	58
		P. gyrina	595	7	10	21
		Oxyloma sp.	3	0	0	0
	SC7 / 2022	*L. stagnalis*	324	11	2	16
		La. elodes	96	0	0	0
		P. gyrina	179	0	1	2
		Oxyloma sp.	16	0	0	0
5	SC2 / 2022	*L. stagnalis*	777	9	12	33
		La. elodes	8	0	0	0
		P. gyrina	337	17	11	32
		Pl. trivolvis	3	0	0	0
		Oxyloma sp.	1	0	0	0

Continued on next page

Table S2 – *Continued from previous page*

Snail richness	Site / Year	Snails collected		Generalist infections	Specialist infections	Total infections
5 (continued)	SC5 / 2020	*L. stagnalis*	115	2	1	4
		La. elodes	15	0	0	0
		P. gyrina	105	0	1	6
		Pl. trivolvis	39	0	0	1
		Oxyloma sp.	26	0	0	0
	SC5 / 2022	*L. stagnalis*	86	1	1	3
		La. elodes	2	0	0	0
		P. gyrina	18	2	0	2
		Pl. trivolvis	17	0	1	1
		Oxyloma sp.	2	0	0	0
	SC6 / 2020	*L. stagnalis*	163	16	6	26
		La. elodes	147	0	1	4
		P. gyrina	230	0	5	6
		Pl. trivolvis	41	0	7	9
		Oxyloma sp.	19	0	0	0
	SC6 / 2021	*L. stagnalis*	360	27	15	46
		La. elodes	55	0	2	4
		P. gyrina	199	0	9	10
		Pl. trivolvis	27	1	8	9
		Oxyloma sp.	13	0	0	0
	SC6 / 2022	*L. stagnalis*	514	9	11	26
		La. elodes	118	0	2	2
		P. gyrina	382	1	1	5
		Pl. trivolvis	2	0	0	0
		Oxyloma sp.	26	0	0	0
		Aplexa sp. [a]	198	0	0	0
	SC7 / 2019	*L. stagnalis*	262	44	5	52
		La. elodes	276	2	5	14
		P. gyrina	287	9	9	22
		Pl. trivolvis	37	5	4	13
		Oxyloma sp.	1	0	0	0
	SC7 / 2020	*L. stagnalis*	335	66	11	112
		La. elodes	13	0	0	2
		P. gyrina	154	2	4	11
		Pl. trivolvis	80	0	12	23
		Oxyloma sp.	8	0	0	0
	SC7 / 2021	*L. stagnalis*	242	18	10	30
		La. elodes	5	0	0	0
		P. gyrina	3	0	0	0
		Pl. trivolvis	13	2	3	5
		Oxyloma sp.	29	0	0	0

[a] *Aplexa* sp. was omitted from the snail richness values in the models so as not to overstate any relationship between increased snail richness and infection prevalence.

Table S3. Results of the multilevel generalized linear mixed effects models examining the relationship between the interaction of snail richness and collection year and overall infection prevalence using a binomial distribution. Odds ratios (OR) with 95% confidence intervals (CI) are presented, along with p-values for the fixed effects. Likelihood ratio test (LRT) p-values indicate the significance of each model component.

Variable	OR (95% CI)	Wald P-value	LRT P-value
**Snail richness**			< 0.001
2	Referent	-	
3	0.41 (0.26-0.65)	< 0.001	
4	1.41 (0.88-2.25)	0.155	
5	1.33 (0.96-1.86)	0.090	
Year			< 0.001
2019	Referent	-	
2020	0.07 (0.03-0.15)	< 0.001	
2021	0.34 (0.21-0.55)	< 0.001	
2022	0.30 (0.20-0.44)	< 0.001	
**Interaction of snail richness and year**			< 0.001
Snail richness 2 * 2019	Referent	-	
Snail richness 3 * 2020	16.78 (7.69-36.60)	< 0.001	
Snail richness 5 * 2020	27.41 (13.46-55.70)	< 0.001	
Snail richness 3 * 2021	23.25 (10.49-51.42)	< 0.001	
Snail richness 4 * 2021	2.04 (1.16-3.60)	0.013	
Snail richness 5 * 2021	4.41 (2.80-6.89)	< 0.001	
Snail richness 4 * 2022	1.33 (0.80-2.18)	0.271	
**Random intercept**	Variance	Standard Deviation	
**Site**	0.9924	0.9962	

Table S4. Results of the multilevel generalized linear mixed effects models examining the relationship between the interaction of snail richness and collection year and generalist infection prevalence using a binomial distribution. Odds ratios (OR) with 95% confidence intervals (CI) are presented, along with p-values for the fixed effects. Likelihood ratio test (LRT) p-values indicate the significance of each model component.

Variable	OR (95% CI)	Wald P-value	LRT P-value
**Snail richness**			< 0.001
2	Referent	-	
3	0.35 (0.17-0.71)	0.004	
4	1.32 (0.63-2.80)	0.464	
5	2.24 (1.31-3.85)	0.003	
Year			< 0.001
2019	Referent	-	
2020	0.05 (0.02-0.17)	< 0.001	
2021	0.31 (0.15-0.65)	0.002	
2022	0.25 (0.13-0.48)	< 0.001	
**Interaction of snail richness and year**			< 0.001
Snail richness 2 * 2019	Referent	-	
Snail richness 3 * 2020	11.41 (3.25-40.04)	< 0.001	
Snail richness 5 * 2020	27.46 (9.02-83.10)	< 0.001	
Snail richness 3 * 2021	15.48 (4.67-51.42)	< 0.001	
Snail richness 4 * 2021	2.01 (0.84-4.81)	0.114	
Snail richness 5 * 2021	4.46 (2.20-9.03)	< 0.001	
Snail richness 4 * 2022	1.90 (0.88-4.10)	0.102	
**Random intercept**	Variance	Standard Deviation	
**Site**	1.7230	1.3130	

Table S5. Heat map of the eight generalist trematode species by site and year. Prevalence was calculated for each generalist species over the identified infections (ID infections) within each site-year; light to dark blue indicates low to high prevalence. Total generalist indicates the total number of generalist infections. *A. burti* = *Australapatemon burti*; *A.* sp. Lin 9C = *Australapatemon* sp. Lineage 9C; *Ec.* sp. A = *Echinoparyphium* sp. A; *E. trivolvis* = *Echinostoma trivolvis* complex Lineage A; *P.* sp. Lin 1 = *Plagiorchis* sp. Lineage 1; *P.* sp. Lin 7 = *Plagiorchis* sp. Lineage 7; Psilo. gen. sp. A = Psilostomidae gen. sp. A; *T. szidati* = *Trichobilharzia szidati.* "-" indicates no infections observed from the relevant trematode.

Site	Year	Snail richness	Total infections	ID infections	Total generalist	*A. burti*	*A.* sp. Lin 9C	*Ec.* sp. A	*E. trivolvis*	*P.* sp. Lin 1	*P.* sp. Lin 7	Psilo. gen. sp. A	*T. szidati*
SC1	2019	4	125	90	76	-	8.9%	1.1%	-	-	40.0%	-	34.4%
	2020	3	23	19	6	-	5.3%	26.3%	-	-	-	-	-
	2021	2	25	21	10	-	19.0%	4.8%	-	-	14.3%	-	9.5%
	2022	4	18	15	10	-	-	20.0%	-	-	33.3%	-	13.3%
SC2	2019	3	88	79	23	21.5%	-	-	-	7.6%	-	-	-
	2020	3	91	68	16	1.5%	-	1.5%	-	19.1%	1.5%	-	-
	2021	4	30	24	3	-	4.2%	4.2%	4.2%	-	-	-	-
	2022	5	65	49	26	-	-	32.7%	-	-	2.0%	-	18.4%
SC3	2019	3	4	4	1	-	-	-	-	-	-	-	25.0%
	2020	2	1	0	0	-	-	-	-	-	-	-	-
	2021	4	109	85	77	-	-	-	-	-	89.4%	-	1.2%
	2022	4	67	51	48	-	-	23.5%	-	-	70.6%	-	-
SC4	2019	3	160	103	72	-	5.8%	8.7%	-	-	52.4%	1.0%	1.9%
	2020	2	10	9	5	-	-	-	-	-	55.6%	-	-
	2021	2	81	59	48	-	1.7%	-	3.4%	-	70.6%	-	18.6%
	2022	2	73	55	45	-	-	5.5%	-	-	70.1%	-	5.5%
SC5	2019	4	10	5	1	-	20.0%	-	-	-	-	-	-
	2020	5	11	4	2	-	-	-	-	-	50.0%	-	-
	2021	3	19	10	5	-	10.0%	-	-	-	30.0%	-	10.0%
	2022	5	6	5	3	-	-	40.0%	-	-	20.0%	-	-

Continued on next page

Table S5 – *Continued from previous page*

Site	Year	Snail richness	Total infections	ID infections	Total generalist	*A. burti*	*A.* sp. Lin 9C	*Ec.* sp. A	*E. trivolvis*	*P.* sp. Lin 1	*P.* sp. Lin 7	Psilo. gen. sp. A	*T. szidati*
SC6	2019	4	85	60	14	6.7%	-	11.7%	-	5.0%	-	-	-
	2020	5	45	35	16	-	14.3%	-	-	-	11.4%	-	20.0%
	2021	5	69	62	28	-	8.1%	-	1.6%	-	17.7%	-	17.7%
	2022	5	33	24	10	4.2%	4.2%	-	-	-	20.8%	-	12.5%
SC7	2019	5	101	83	60	2.4%	10.8%	12.1%	-	1.2%	3.6%	3.6%	38.6%
	2020	5	148	95	68	-	27.4%	1.1%	-	-	28.4%	-	14.7%
	2021	5	35	33	20	-	15.2%	3.0%	3.0%	-	27.3%	-	12.1%
	2022	4	18	14	11	-	-	-	-	-	21.4%	-	57.1%
SC8	2019	3	84	82	64	-	1.2%	2.4%	3.7%	-	48.8%	19.5%	2.4%
	2020	3	15	10	2	10.0%	10.0%	-	-	-	-	-	-
	2021	3	2	2	2	-	-	-	-	-	50.0%	-	50.0%
	2022	2	135	88	74	-	5.7%	-	-	-	22.7%	-	55.7%
**Total**			**1 786**	**1 343**	**846**	**1.9%**	**6.0%**	**5.6%**	**0.6%**	**1.7%**	**31.2%**	**1.5%**	**14.5%**

Table S6. Summary of the generalist trematode heat map results presented in Table S5. High snail richness includes snail richness 4 and 5; low snail richness includes snail richness 2 and 3. Prev. = prevalence.

Species	Mean prev. high snail richness	Mean prev. low snail richness
Australapatemon burti	4.4%	11.0%
Australapatemon sp. Lineage 9C	12.6%	7.3%
Echinoparyphium sp. A	14.9%	8.2%
Echinostoma trivolvis complex Lineage A	2.9%	3.6%
Plagiorchis sp. Lineage 1	3.1%	13.4%
Plagiorchis sp. Lineage 7	31.1%	41.6%
Psilostomidae gen sp. A	3.6%	10.2%
Trichobilharzia szidati	21.8%	19.8%

Table S7. Results of the multilevel generalized linear mixed effects models examining the relationship between snail richness and collection year and specialist infection prevalence using a binomial distribution. Odds ratios (OR) with 95% confidence intervals (CI) are presented, along with p-values for the fixed effects. Likelihood ratio test (LRT) p-values indicate the significance of each model component.

Variable	OR (95% CI)	Wald P-value	LRT P-value
**Snail richness**			0.09
2	Referent	-	
3	1.75 (1.12-2.75)	0.015	
4	1.80 (1.07-3.00)	0.026	
5	1.68 (1.03-2.75)	0.039	
**Year**			< 0.001
2019	Referent	-	
2020	1.26 (0.97-1.63)	0.081	
2021	1.89 (1.39-2.59)	< 0.001	
2022	0.47 (0.33-0.67)	< 0.001	
**Random intercept**	Variance	Standard Deviation	
**Site**	0.6421	0.8013	

Table S8. Average maximum temperature (°C) data for the months June, July, and August for sampling years 2019-2022. Data recorded at the Oliver AGDM station (coordinates: 53.650000, -113.350000) (Environment and Climate Change Canada, 2025). One data point was missing in August 2021 (August 10), and two were missing in June 2022 (June 5 and June 12).

Month	Year	Average maximum temperature	Standard deviation
June	2019	19.58	4.28
July	2019	21.95	3.21
August	2019	20.13	3.79
June	2020	20.01	3.89
July	2020	22.84	2.97
August	2020	22.55	3.92
June	2021	24.56	5.93
July	2021	25.65	5.51
August	2021	23.55	5.23
June	2022	21.18	2.63
July	2022	24.15	3.79
August	2022	26.09	3.91

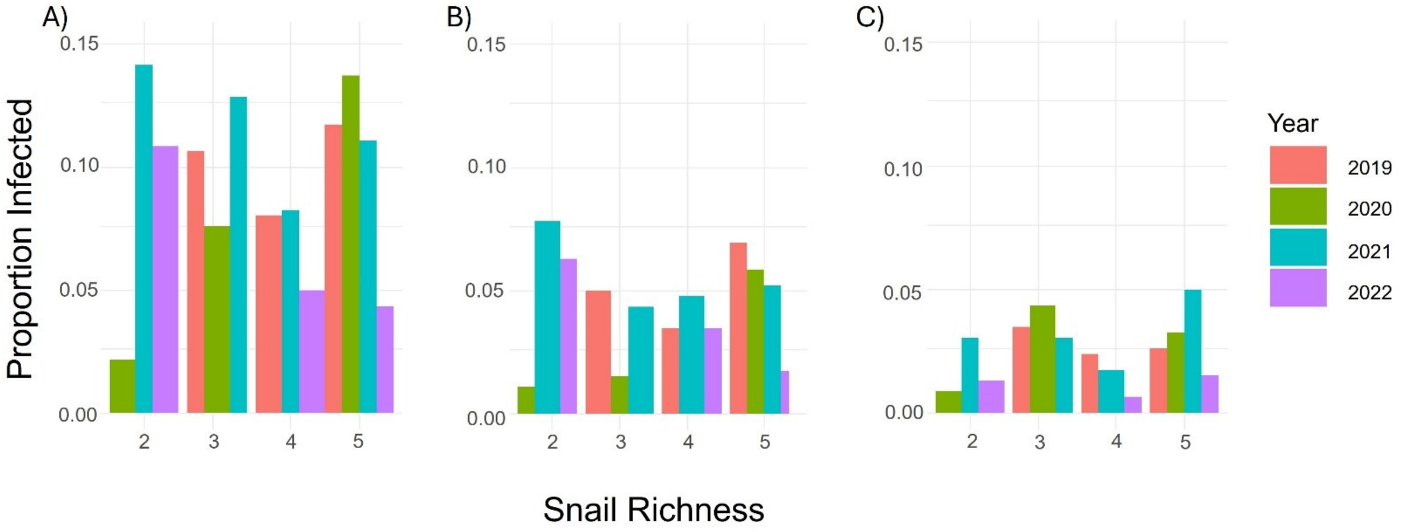

Figure S1. The proportion of infected snails grouped by snail richness level. A) Proportion of snails infected with a trematode. Data includes all infected snails, with or without high-quality DNA sequence data. B) Proportion of snails infected with a generalist trematode by snail richness level and year. C) Proportion of snails infected with a specialist trematode by snail richness level and year.

A)

	ORs (95% CIs)				
	Snail richness 3				
	Year	2019	2020	2021	2022
Snail richness 2	**2019**	NA	NA	NA	NA
	**2020**	0.17 (0.06-0.49)	0.14 (0.05-0.44)	0.02 (0.01-0.09)	NA
	**2021**	0.82 (0.53-1.27)	0.69 (0.38-1.22)	0.10 (0.04-0.30)	NA
	**2022**	0.71 (0.51-1.01)	0.60 (0.35-1.01)	0.09 (0.03-0.25)	NA

B)

	ORs (95% CIs)				
	Snail richness 4				
	Year	2019	2020	2021	2022
Snail richness 2	**2019**	NA	NA	NA	NA
	**2020**	0.05 (0.02-0.16)	NA	0.07 (0.02-0.23)	0.13 (0.04-0.40)
	**2021**	0.24 (0.13-0.45)	NA	0.35 (0.18-0.67)	0.62 (0.32-1.18)
	**2022**	0.21 (0.12-0.38)	NA	0.30 (0.17-0.56)	0.54 (0.29-0.99)

C)

	ORs (95% CIs)				
	Snail richness 5				
	Year	2019	2020	2021	2022
Snail richness 2	**2019**	NA	NA	NA	NA
	**2020**	0.05 (0.02 - 0.18)	0.03 (0.01-0.09)	0.04 (0.01-0.12)	0.18 (0.06-0.56)
	**2021**	0.25 (0.12-0.55)	0.13 (0.07-0.26)	0.17 (0.08-0.35)	0.86 (0.47-1.59)
	**2022**	0.22 (0.11-0.47)	0.11 (0.06-0.22)	0.15 (0.07-0.30)	0.75 (0.43-1.31)

Figure S2. Odds ratios (OR) followed by 95% confidence intervals comparing snail richness levels across study years for the overall infection model. The data included are all snails infected with a digenetic trematode across the indicated year. Data that were non-estimable are denoted as NA. Gray cells indicate significant p-values < 0.05. ORs < 1.0 indicate that the prevalence was lower at a snail richness of 2. A) Snail richness = 2 compared to snail richness = 3. B) Snail richness = 2 compared to snail richness = 4. C) Snail richness = 2 compared to snail richness = 5.

A)

		ORs (95% CIs)			
		Snail richness 3			
	Year	2019	2020	2021	2022
Snail richness 2	2019	NA	NA	NA	NA
	2020	0.16 (0.03-0.74)	0.25 (0.04-1.42)	0.03 (0.004-0.27)	NA
	2021	0.88 (0.50-1.56)	1.42 (0.54-3.75)	0.18 (0.04-0.88)	NA
	2022	0.72 (0.46-1.13)	1.15 (0.45-2.93)	0.15 (0.03-0.66)	NA

B)

		ORs (95% CIs)			
		Snail richness 4			
	Year	2019	2020	2021	2022
Snail richness 2	2019	NA	NA	NA	NA
	2020	0.04 (0.01-0.23)	NA	0.07 (0.01-0.35)	0.09 (0.02-0.46)
	2021	0.23 (0.09-0.58)	NA	0.38 (0.15-0.92)	0.49 (0.20-1.17)
	2022	0.19 (0.08-0.47)	NA	0.31 (0.13-0.71)	0.40 (0.17-0.92)

C)

		ORs (95% CIs)			
		Snail richness 5			
	Year	2019	2020	2021	2022
Snail richness 2	2019	NA	NA	NA	NA
	2020	0.02 (0.004-0.15)	0.02 (0.003-0.10)	0.02 (0.003-0.11)	0.10 (0.02-0.53)
	2021	0.14 (0.05-0.42)	0.09 (0.03-0.26)	0.10 (0.03-0.29)	0.55 (0.21-1.41)
	2022	0.11 (0.04-0.33)	0.08 (0.03-0.20)	0.08 (0.03-0.23)	0.45 (0.18-1.11)

Figure S3. Odds ratios (OR) followed by 95% confidence intervals comparing richness levels across study years for the generalist infection model. Data included are trematode infections that were identified using DNA sequencing and determined to be generalist trematodes based on the Paired Differences Index calculated using the bipartite package (Dormann et al., 2024), see section 2.21 for more details. Data that were non-estimable are denoted as NA. Gray cells indicate significant p-values < 0.05. ORs < 1.0 indicate that the prevalence was lower at a snail richness of 2. A) Snail richness = 2 compared to snail richness = 3. B) Snail richness = 2 compared to snail richness = 4. C) Snail richness = 2 compared to snail richness = 5.

All years	ORs (95% CIs)			
Snail richness	2	3	4	5
2		0.57 (0.36-0.90)	0.56 (0.33-0.93)	0.60 (0.37-0.97)
3			0.97 (0.67-1.41)	1.04 (0.69-1.58)
4				1.07 (0.78-1.48)
5				

Figure S4. Odds ratios (OR) followed by 95% confidence intervals comparing richness levels across study years for the specialist infection model. Data included are trematode infections that were identified using DNA sequencing and determined to be specialist trematodes based on the Paired Differences Index calculated using the bipartite package (Dormann et al., 2024), see section 2.21 for more details. The richness values in the rows are first in the comparisons. ORs < 1.0 indicate that the prevalence was lower for the first richness in the comparison.

		ORs (95% CIs)			
	2022				
2019	Snail richness	2	3	4	5
	2	NA	NA	NA	NA
	3	1.40 (0.99-1.98)	NA	0.75 (0.44-1.28)	1.05 (0.66-1.67)
	4	4.76 (2.62-8.67)	NA	2.56 (1.57-4.16)	3.58 (2.14-5.99)
	5	4.52 (2.15-9.48)	NA	2.42 (1.35-4.35)	3.39 (1.73-6.62)

Figure S5. Odds ratios (ORs) and 95% confidence intervals for trematode infection prevalence at each snail richness level for the years 2019 (first year of snail sampling) and 2022 (last year of snail sampling) using the overall infection model. Data used are all snails infected with a trematode in 2019 and 2022. Data that were non-estimable are denoted as NA. Grey cells indicate comparisons that were significant (p-values < 0.05). ORs > 1.0 indicate that the prevalence of infection with a digenetic trematode was greater in 2019 compared to 2022 at the relevant snail richness level.

A)

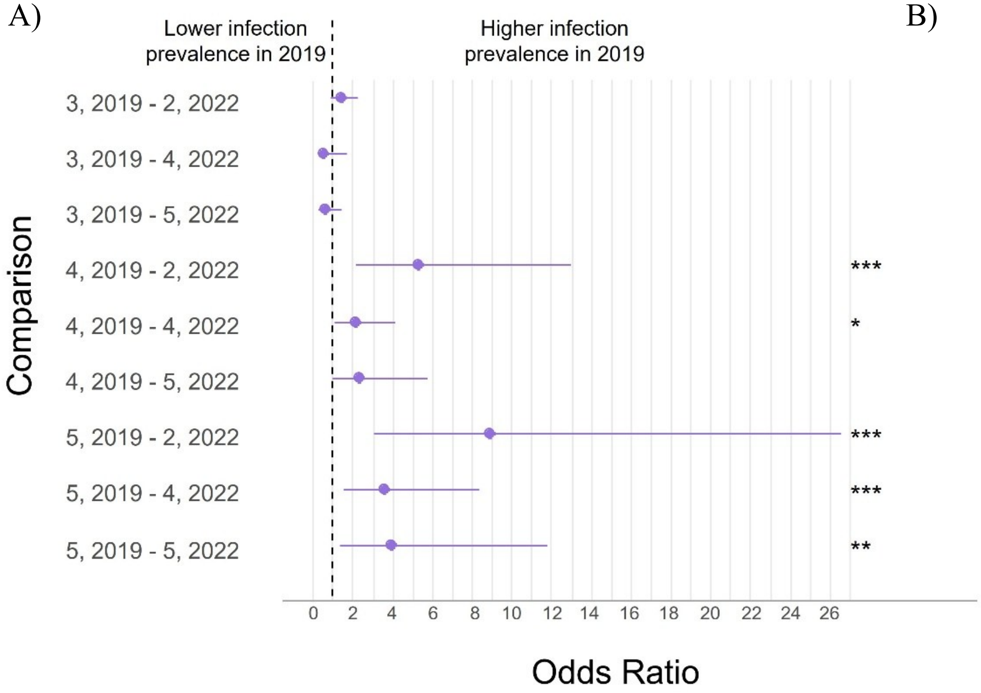

B)

		ORs (95% CIs)			
	2022				
	Snail richness	2	3	4	5
2019	2	NA	NA	NA	NA
	3	1.40 (0.89-2.19)	NA	0.56 (0.26-1.67)	0.62 (0.28-1.38)
	4	5.27 (2.14-12.94)	NA	2.10 (1.06-4.13)	2.35 (0.97-5.70)
	5	8.93 (3.00-26.54)	NA	3.55 (1.51-8.34)	3.98 (1.34-11.82)

Figure S6. A) Plot showing the estimable Odds ratios (ORs) with 95% confidence intervals (CIs) for trematode infection prevalence at each snail richness level for the year 2022 (last year of snail sampling) compared to 2019 (first year of snail sampling) using the generalist infection model. ORs > 1.0 indicate that the prevalence of infection with a digenetic trematode was greater in 2019 than in 2022 at the indicated snail richness level. Comparisons are labelled as "snail richness, year – snail richness, year," where the first snail richness is the reference. * $p < 0.05$; ** $p < 0.01$; *** $p \leq 0.0001$. B) All pairwise comparisons, showing ORs with 95% CIs for trematode infection prevalence between 2019 and 2022 at each snail richness level. Comparisons that were non-estimable are denoted as NA. Grey cells indicate comparisons that were significant (p-values < 0.05).

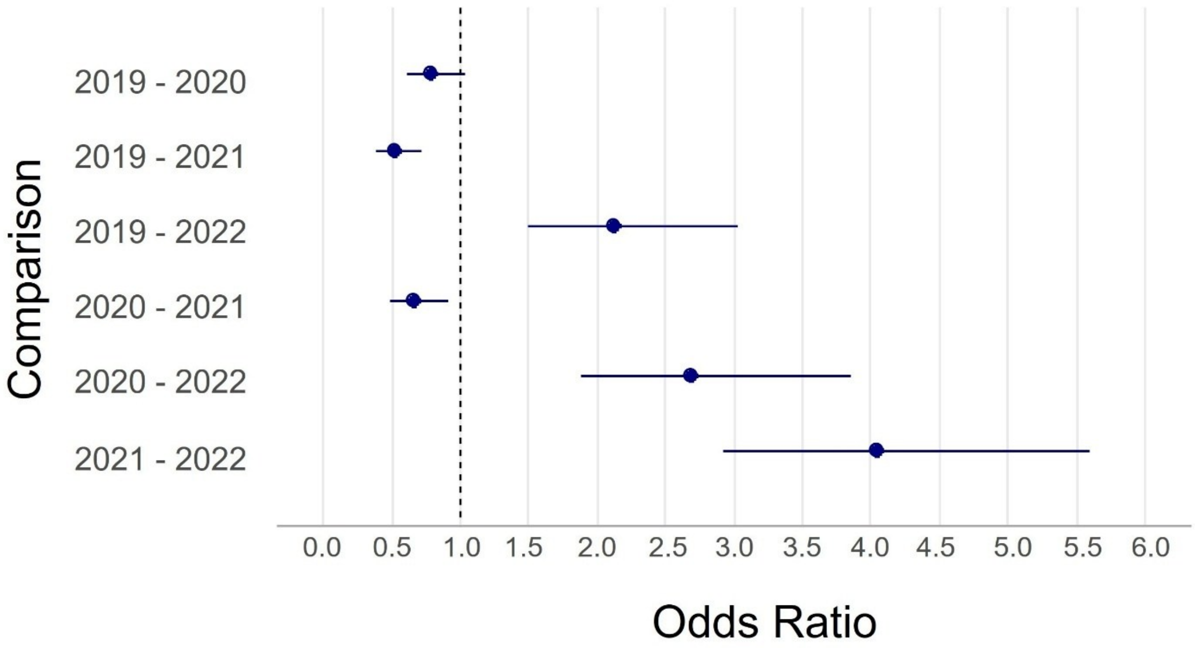

Figure S7. Plot showing the estimable Odds ratios (ORs) with 95% confidence intervals (CIs) comparing infection prevalence across years for the specialist infection model. Comparisons are labelled as “year - year,” where the first year is the reference. ORs > 1.0 indicate that the prevalence of infection with a digenetic trematode was greater in the reference year. This plot is presented to visualize trends only, p-values not reported because snail richness was not significant in the specialist model (see Table S5).

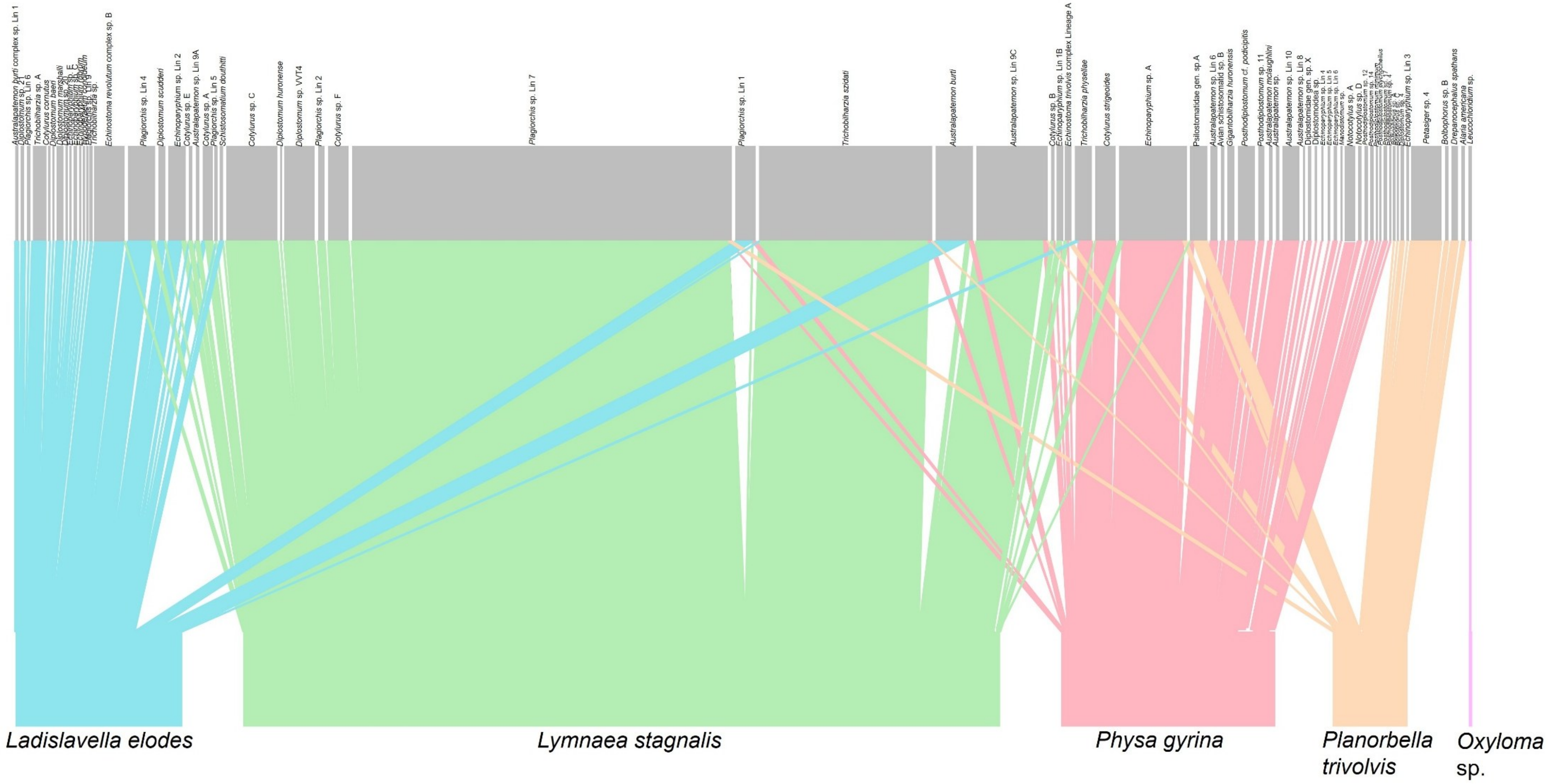

Figure S8. Quantitative network diagram of snail-trematode relationships observed across all study sites and years. The snail hosts are found along the bottom, and the trematode species are indicated at the top. Relationships shown indicate the number of times each snail-trematode relationship was observed. Created using the package bipartite (Dormann et al., 2024).

Literature Cited in Supporting Information

Dormann, C. F., Fruend, J., & Gruber, B. (2024). *bipartite: Visualising bipartite networks and calculating some (ecological) indices*. https://doi.org/10.32614/CRAN.package.bipartite

Environment and Climate Change Canada. (2025). *Historical Data*. https://climate.weather.gc.ca/historical_data/search_historic_data_e.html
